# Machine learning-enabled maternal risk assessment for women with pre-eclampsia (the PIERS-ML model): a modelling study

**DOI:** 10.1016/S2589-7500(23)00267-4

**Published:** 2024-03-20

**Authors:** Tünde Montgomery-Csobán, Kimberley Kavanagh, Paul Murray, Chris Robertson, Sarah J E Barry, U Vivian Ukah, Beth A Payne, Kypros H Nicolaides, Argyro Syngelaki, Olivia Ionescu, Ranjit Akolekar, Jennifer A Hutcheon, Laura A Magee, Peter von Dadelszen, PIERS Consortium, Mark A. Brown, Gregory K. Davis, Claire Parker, Barry N. Walters, Nelson Sass, J. Mark Ansermino, Vivien Cao, Geoffrey W. Cundiff, Emma C.M. von Dadelszen, M. Joanne Douglas, Guy A. Dumont, Dustin T. Dunsmuir, Jennifer A. Hutcheon, K.S. Joseph, Sayrin Lalji, Tang Lee, Jing Li, Kenneth I. Lim, Sarka Lisonkova, Paula Lott, Jennifer M. Menzies, Alexandra L. Millman, Lynne Palmer, Beth A. Payne, Ziguang Qu, James A. Russell, Diane Sawchuck, Dorothy Shaw, D. Keith Still, U. Vivian Ukah, Brenda Wagner, Keith R. Walley, Dany Hugo, The late Andrée Gruslin, George Tawagi, Graeme N. Smith, Anne-Marie Côté, Jean-Marie Moutquin, Annie B. Ouellet, Shoo K. Lee, Tao Duan, Jian Zhou, The late Farizah Haniff, Swati Mahajan, Amanda Noovao, Hanna Karjalainend, Alja Kortelainen, Hannele Laivuori, J. Wessel Ganzevoort, Henk Groen, Phillipa M. Kyle, M. Peter Moore, Barbra Pullar, Zulfiqar A. Bhutta, Rahat N. Qureshi, Rozina Sikandar, The late Shereen Z. Bhutta, Garth Cloete, David R. Hall, The late Erika van Papendorp, D. Wilhelm Steyn, Christine Biryabarema, Florence Mirembe, Annettee Nakimuli, John Allotey, Shakila Thangaratinam, Kypros H. Nicolaides, Olivia Ionescu, Argyro Syngelaki, Michael de Swiet, Laura A. Magee, Peter von Dadelszen, Ranjit Akolekar, James J. Walker, Stephen C. Robson, Fiona Broughton-Pipkin, Pamela Loughna, Manu Vatish, Christopher W.G. Redman, Sarah J.E. Barry, Kimberley Kavanagh, Tunde Montgomery-Csobán, Paul Murray, Chris Robertson, Eleni Z. Tsigas, Douglas A. Woelkers, Marshall D. Lindheimer, William A. Grobman, Baha M. Sibai, Mario Merialdi, Mariana Widmer

**Affiliations:** a Department of Mathematics and Statistics, University of Strathclyde, Glasgow, UK; b Department of Electronic and Electrical Engineering, University of Strathclyde, Glasgow, UK; c Department of Epidemiology, Biostatistics, and Occupational Health, McGill University, Montréal, QC, Canada; d School of Population and Public Health, University of British Columbia, Vancouver, BC, Canada; e Department of Obstetrics and Gynaecology, University of British Columbia, Vancouver, BC, Canada; f Institute of Women and Children's Health, University of British Columbia, Vancouver, BC, Canada; g Harris Birthright Research Centre for Fetal Medicine, King's College Hospital, London, UK; h Fetal Medicine Unit, Medway Maritime Hospital, Gillingham, UK; i Institute of Medical Sciences, Canterbury Christ Church University, Chatham, UK; j Department of Women and Children's Health, School of Life Course and Population Sciences, King's College London, London UK

## Abstract

**Background:**

Affecting 2–4% of pregnancies, pre-eclampsia is a leading cause of maternal death and morbidity worldwide. Using routinely available data, we aimed to develop and validate a novel machine learning-based and clinical setting-responsive time-of-disease model to rule out and rule in adverse maternal outcomes in women presenting with pre-eclampsia.

**Methods:**

We used health system, demographic, and clinical data from the day of first assessment with pre-eclampsia to predict a Delphi-derived composite outcome of maternal mortality or severe morbidity within 2 days. Machine learning methods, multiple imputation, and ten-fold cross-validation were used to fit models on a development dataset (75% of combined published data of 8843 patients from 11 low-income, middle-income, and high-income countries). Validation was undertaken on the unseen 25%, and an additional external validation was performed in 2901 inpatient women admitted with pre-eclampsia to two hospitals in south-east England. Predictive risk accuracy was determined by area-under-the-receiver-operator characteristic (AUROC), and risk categories were data-driven and defined by negative (–LR) and positive (+LR) likelihood ratios.

**Findings:**

Of 8843 participants, 590 (6·7%) developed the composite adverse maternal outcome within 2 days, 813 (9·2%) within 7 days, and 1083 (12·2%) at any time. An 18-variable random forest-based prediction model, PIERS-ML, was accurate (AUROC 0·80 [95% CI 0·76–0·84] *vs* the currently used logistic regression model, fullPIERS: AUROC 0·68 [0·63–0·74]) and categorised women into very low risk (–LR <0·1; eight [0·7%] of 1103 women), low risk (–LR 0·1 to 0·2; 321 [29·1%] women), moderate risk (–LR >0·2 and +LR <5·0; 676 [61·3%] women), high risk (+LR 5·0 to 10·0, 87 [7·9%] women), and very high risk (+LR >10·0; 11 [1·0%] women). Adverse maternal event rates were 0% for very low risk, 2% for low risk, 5% for moderate risk, 26% for high risk, and 91% for very high risk within 48 h. The 2901 women in the external validation dataset were accurately classified as being at very low risk (0% with outcomes), low risk (1%), moderate risk (4%), high risk (33%), or very high risk (67%).

**Interpretation:**

The PIERS-ML model improves identification of women with pre-eclampsia who are at lowest and greatest risk of severe adverse maternal outcomes within 2 days of assessment, and can support provision of accurate guidance to women, their families, and their maternity care providers.

**Funding:**

University of Strathclyde Diversity in Data Linkage Centre for Doctoral Training, the Fetal Medicine Foundation, The Canadian Institutes of Health Research, and the Bill & Melinda Gates Foundation.

## Introduction

Complicating 2–4% of pregnancies, pre-eclampsia (defined as new-onset hypertension at or after 20 weeks’ gestation, accompanied by either new-onset proteinuria, other maternal target organ damage, or evidence of uteroplacental dysfunction)^1^, ^2^ remains a leading global cause of maternal mortality and life-threatening morbidity.^1^, ^2^, ^3^, ^4^, ^5^ More than 99% of the annual 46 000 pre-eclampsia-related maternal deaths occur in low-income and middle-income countries (LMICs).^6^

In pregnancies complicated by pre-eclampsia, it is clear that perinatal survival without major morbidity is largely related to gestational age at birth.^7^ However, the burden of adverse maternal outcomes is spread across gestation. Although maternal risks are proportionately greater with the earlier onset of pre-eclampsia,^5^, ^8^, ^9^ the population level burden of maternal risk is borne by the 75–80% of cases of pre-eclampsia that arise at term.^10^

The sole method of initiating recovery from pre-eclampsia is delivery of the placenta.^2^ At term, the focus is initiating birth.^11^ Before term, women and their maternity care providers balance maternal risks from evolving disease with prematurity-related perinatal risks by subjectively integrating ongoing assessments of symptoms, signs, and laboratory tests.^2^ In busy maternity units, considerable experience informs decisions; however, most women with preterm pre-eclampsia are managed, at least initially, by relatively inexperienced maternity care providers. For many LMICs and disadvantaged high-income country populations, access to comprehensive obstetric and newborn care is limited.

**Research in context**

**Evidence before this study**

Pre-eclampsia is associated with increased risks of maternal morbidity and mortality. A systematic review published in January, 2018, summarised previous studies presenting predictive models of adverse outcomes of pre-eclampsia. These studies included univariable models and multivariable models using logistic or Cox regression. Of the reviewed models, the fullPIERS logistic regression model was identified as having the best performance. On Jan 19, 2024, we searched PubMed using the search terms (“outcome”) and (“preeclampsia” or “pre-eclampsia”), and (“model” or “risk” or “algorithm”) in the title published since the 2018 review paper (from Jan 1, 2018, to Jan 19, 2024) and found a further seven studies presenting models of adverse maternal outcomes of pre-eclampsia from single country studies in Zimbabwe, Germany, and China. Participant numbers ranged from 319 to 2532, and the studies each collected data from one or two institutions. All except one of the models predicted a combined maternal and neonatal outcome. Of the seven studies, four studies created logistic regression models, one study created a machine learning model, and two studies created both logistic regression and machine learning models. Of the machine learning models, two models were created based on low-income or middle-income country data. A single high-income country institution, artificial intelligence-based model has been developed that predicts a combined adverse maternal and perinatal outcome. No model was reported to have better performance than the fullPIERS model, and only one model was externally validated, with poor performance.

**Added value of this study**

We recruited 8843 women from 53 maternity units in 11 low-income, middle-income countries, and high-income countries to develop and validate the PIERS-ML model using a random forest method, and 2901 women to externally validate the model. Maternal risk strata were defined by diagnostic test performance criteria (likelihood ratios) for variables collected within 24 h of admission with pregnancy hypertension in predicting the occurrence of any element of a combined adverse maternal outcome within 48 h, with useful performance at 7 days and any time after admission. In addition, the PIERS-ML model identified women at very low risk of experiencing eclampsia and stillbirth. This performance was repeated in the external validation cohort as expected.

**Implications of all the available evidence**

The PIERS-ML model, to our knowledge, quantifies maternal risks in women with pre-eclampsia and provides useful risk stratification to guide joint decision making between clinicians and patients about place of care (eg, in utero transfer to higher levels of care), co-interventions (eg, antenatal corticosteroids and magnesium sulphate), and timing of birth (eg, labour induction and caesarean birth). A dynamic modelling extension of PIERS-ML would facilitate ongoing care beyond initial assessment. New variables (eg, angiogenic markers) should be tested to determine their possible inclusion in future versions of the model.

To optimise maternal outcomes in pre-eclampsia, we need objective, time-of-disease maternal risk assessment to inform decision making during the following 48 h, wherever that woman lives. We previously used logistic regression to develop a model—fullPIERS (pre-eclampsia integrated estimate of risk)—for high-income countries.^8^ In this study, we aimed to harness the strengths of machine learning-based classifiers to test the hypothesis that it is possible to develop and externally validate a novel globally-relevant PIERS-ML model using information routinely-available at presentation with pre-eclampsia.

## Methods

### Study design and included datasets

For this machine leaning model (PIERS-ML), we used prospectively collected data from women with pre-eclampsia, broadly-defined according to the 2021 International Society for the Study of Hypertension in Pregnancy criteria,^1^ as “women presented for initial facility-based assessment at centres with general policies of expectant management of pre-eclampsia remote from term”.

To maximise the sample size for machine learning, data were collated from published model development and validation studies for the miniPIERS model (2008–12; N=2126 from Brazil, Fiji, Pakistan, South Africa, and Uganda)^5^ and development and validation for the fullPIERS model (2003–16; N=6717 from Australia, Canada, Finland, New Zealand, UK, and USA).^8^, ^12^ A randomly assigned 75·0% (6633 of 8843 women) of the combined cohort was used for model development, 12·5% (1107 women) were used to select thresholds for risk strata, and 12·5% (1103 women) were reserved for model validation, which was completely unseen by the model during training. This individual participant data meta-analysis was prospectively registered with PROSPERO (CRD42020195616).

The model was externally validated on the second dataset from a prospective observational cohort study using the electronic health records of 2901 women with singleton pregnancies who were admitted with a diagnosis of pre-eclampsia (using the definition from the International Society for the Study of Hypertension in Pregnancy^1^) to King's College Hospital, London, UK, and Medway Maritime Hospital, Gillingham, UK, between Dec 1, 2013, and Dec 31, 2021. All women whose data were used in the external validation dataset gave written informed consent to participate in the study, which was conducted according to the guidelines of the Declaration of Helsinki and approved by the NHS Research Ethics Committee (REC reference: 02-03-033).

### Study variables

Variables were considered for modelling only if assessed before the occurrence of any component of the combined adverse maternal outcome. Variables detailed the woman's health system, her demographics, past and current medical and obstetric history, and relevant symptoms, signs, and laboratory tests. For face validity, at least one objective variable was required for each cardiorespiratory, renal, hepatic, and haematological organ system.

### Outcomes

The primary study outcome was a composite developed by the Delphi consensus^13^ and defined as the first occurrence of one or more maternal mortality or severe maternal morbidity (appendix pp 5–6), within 2 days of first assessment for pre-eclampsia.

### Missing variables and machine learning

The most clinically relevant abnormal value of each variable obtained during assessment on the first day of admission was taken to create a dataset, with only one observation per woman per day. The most clinically relevant abnormal value was defined as a positive response for any of the symptom variables, the minimum value for oxygen saturation, platelet count, fibrinogen, serum albumin, and random glucose, and the maximum value for all other repeated variables (data missingness is detailed in the appendix pp 8–9). Variables were excluded if at least 60% of values were missing.^14^, ^15^ Variables with a lower proportion of missing values were included in multiple imputation if values were missing at random (ie, the value of other variables could explain why something was missing), or completely at random (ie, has no explanation). As 18·6% of all data were missing (64 262 missing values of 344 877 values), development and validation datasets were imputed 20 times each (number of imputations equal to our greater than percentage of incomplete cases), using chained random forests (appendix p 18). Models were fitted on each development dataset (random 75% of the data) using ten-fold cross-validation. Multiple machine learning methods along with feature selection from the initial 33 variables were tested and compared (appendix pp 13–14) with random forest performing best (random forest methodology is explained in the appendix pp 18–19). Models were tested on each imputed validation dataset (12·5% [1103 of 8843 women] of the data), and predictions were combined into a mean prediction per woman. The 18-variable random forest model, where, for parsimony, had variables with above average importance using the Gini index, was chosen as the final model.

### Model testing and validation

We assessed the model's ability to classify women into outcome and no outcome groups, using the remaining 12·5% (1103 of 8843 women) of the data, using the area-under-the-receiver-operator characteristic (AUROC), calibration, and precision-recall curves. Decision curve analysis was also carried out to assess clinical utility (see appendix p 24 for details of model calibration, decision curve analysis, and precision-recall curve). Likelihood ratios used to determine risk strata were data-defined as follows: very low risk (by a negative likelihood ratio <0·10), low risk (negative likelihood ratio of 0·1–0·2), high risk (positive likelihood ratio of 5·0–10·0), very high risk (positive likelihood ratio >10·0), and moderate risk otherwise (appendix pp 19–20).^16^ Positive likelihood ratios for very high and high risk were calculated by splitting the testing data into a very high risk and not very high risk group, and high risk and less than high risk group, and then calculating likelihood ratios for a two-group prediction using sensitivity and specificity.^17^ Similarly, negative likelihood ratios for very low risk and low risk were calculated by creating very low risk and not very low risk group, and low risk and higher than low risk group.^17^

Thereafter, we assessed the stratification accuracy of the PIERS-ML model in the external validation cohort. Following the selection of the variables in the PIERS-ML model, the external validation dataset had 18·6% (64 262 missing values of 344 877 values) missingness. These missing values were imputed 20 times, independently of the existing combined PIERS-ML dataset. The PIERS-ML model was applied to each of the 20 imputed datasets and the mean prediction per woman was taken. Oxygen saturation was not collected from women in the external validation cohort, so it was assumed to be normal and replaced uniformly by 97%, which was the expected normal measurement. Data for some less common components of the combined adverse maternal outcome were not available in women's electronic health records, and some other components were conflated into summary measures.

### Sensitivity analyses

For the sensitivity analyses, new datasets were created in which additional coagulation-related variables were included (as they are costly in all health systems), and mean platelet volume was excluded (as it is not routinely reported by all haematology laboratories). Modelling steps were repeated for these datasets and the results were compared.

Secondary analyses were undertaken to predict the following**:** (1) outcomes at either 7 days or at any time following admission until primary hospital discharge (outcomes within 7 days included patients who had outcomes within 2 days, and outcomes at any time included outcomes within 2 days and 7 days); and (2) outcomes limited to eclampsia or stillbirth.

As some renal and haematological measures were included in both the candidate variables and the definitions of components of the combined maternal outcomes, we assessed the performance of the model in women who experienced no renal, no haematological, or neither renal nor haematological outcomes. The influence of multiple imputation on predictions was assessed by complete case analysis and mean imputation (appendix p 25).

### Comparison with the fullPIERS model

To compare the performance of the PIERS-ML model with the fullPIERS model, predicted probabilities were calculated for the validation dataset using the fullPIERS logistic regression model,^8^ and AUROC, calibration, and stratification were compared with PIERS-ML. As the fullPIERS model must be adjusted for use in a new setting,^18^ the model was refitted on the combined, imputed development dataset by fitting a logistic regression model using the same variables and interactions as the fullPIERS model on each of the imputed datasets using ten-fold cross-validation. The same performance assessment was repeated for the refitted model as for the original.

### Statistical analysis

All analyses were performed using R Studio (version 2022.02.2+492) and multiple imputation was carried out using missRanger (version 2.2.1). Random forests were fitted on each dataset using the caret package (version 6.0-94) with ten-fold cross-validation using the “rf” method, and combined into one ensemble model using the caretEnsemble (version 2.0.2) package. To compare cohort characteristics, χ^2^ and Fisher's exact tests were used for categorical variables, and Mann-Whitney *U* tests were used to were used for continuous variables, with statistical significance set at p<0·05.

### Role of the funding source

The funders of the study had no role in the study design, data collection, data analysis, data interpretation, or writing of the report.

## Results

Data were available for 8843 eligible women with pre-eclampsia, who were recruited from 53 institutions (appendix pp 2–4) in 11 countries (Australia, Brazil, Canada, Fiji, Finland, New Zealand, Pakistan, South Africa, Uganda, UK, and USA), with a median of 1244 (IQR 693·5–1983·5) women per included cohort.^5^, ^8^, ^12^, ^19^, ^20^, ^21^ Other cohort details have been published previously.^5^, ^8^, ^12^ For external validation from two new institutions, data were available from an additional 2901 women, and collected as part of a prospective observational study.

Health system and individual-level characteristics of women who experienced an adverse maternal outcome differed from women who did not (table 1; appendix pp 6–7). Women who experienced adverse outcomes were more often cared for in countries with lower per capita gross domestic products. These women were younger, more likely to have multiple pregnancies, and presented at an earlier gestational age; less often, their past history was complicated by chronic hypertension and their pregnancies by gestational diabetes. The study population had similar proportions of women from White, Asian, or Black ethnic backgrounds, regardless of complications.

**Table 1:** Baseline characteristics, co-interventions, and pregnancy outcomes of the study cohort

	**PIERS-ML group**			**External validation group**		
	Women with an adverse outcome at any time after first assessment (N=1083)	Women without an adverse outcome (N=7760)	p value*	Women with an adverse outcome at any time after first assessment (N=121)	Women without an adverse outcome (N=2780)	p value*
**Health system**						
National per capita gross domestic product (US$)	41 064 (7501–46 594)	43 586 (28 206–50 114)	<0·0001	42 330 (41 064–43 043)	42 330 (40 361–43 043)	0·0427
National maternal mortality ratio (maternal deaths per 100 000 live births)	11 (10–161)	11 (11–15)	0·0693	8 (7·5–8·0)	8 (7·0–8)	0·13
**Demographics**						
Race	..	..	0·0259	Not collected	Not collected	..
White	379 (34·8%)	2485 (31·8%)	..	..	..	..
Asian	323 (29·7%)	2365 (30·2%)	..	..	..	..
Black	303 (27·8%)	2001 (25·6%)	..	..	..	..
Other	119 (10·9%)	909 (11·6%)	..	..	..	..
Maternal age at expected date of delivery (years)	31 (26–35)	31 (27–36)	0·0377	29 (26–34)	31 (27–35)	0·20
Nulliparous	653 (60·0%)	4542 (58·1%)	0·25	..	Not collected	..
Multiple pregnancy	128 (11·8%)	529 (6·8%)	<0·0001	Not collected	Not collected	..
Gestational age at eligibility (weeks)	33·4 (30·0–37·1)	36·0 (32·0–38·4)	<0·0001	35·6 (31·4–38·9)	37·9 (35·6–39·7)	<0·0001
**Past and current medical and obstetrical history**						
Cigarette smoking	139 (12·8%)	1011 (12·9%)	0·9232	Not collected	Not collected	..
Chronic hypertension	146 (13·4%)	1324 (16·9)	0·003	Not collected	Not collected	..
Pre-gestational renal disease	63 (5·8%)	520 (6·6%)	0·30	Not collected	Not collected	..
Pre-gestational diabetes	61 (5·6%)	409 (5·2%)	0·61	Not collected	Not collected	..
Gestational diabetes	75 (6·9%)	974 (12·5%)	<0·0001	Not collected	Not collected	..
**Symptoms on day of first assessment**						
Nausea or vomiting	107 (9·8%)	443 (5·7%)	<0·0001	20 (16·5%)	175 (6·3%)	<0·0001
Headache or visual disturbance	406 (37·3%)	2132 (27·3%)	<0·0001	54 (44·6%)	760 (27·4%)	<0·0001
Right upper quadrant or epigastric pain	205 (18·8%)	754 (9·6%)	<0·0001	27 (22·3%)	138 (5·0%)	<0·0001
Chest pain or dyspnoea	73 (6·7%)	117 (1·5%)	<0·0001	17 (14·1%)	29 (1·0%)	<0·0001
**Signs on day of first assessment**						
Height (cm)	162 (157–166)	163 (157–168)	0·24	164 (160–168)	165 (161–169·6)	0·0689
Weight (kg)	78·0 (68·5–87·0)	81·0 (70·3–93·4)	<0·0001	77·0 (67·5–88)	85·9 (75–100)	<0·0001
Systolic blood pressure (mm Hg)	156 (147–166)	151 (140–161)	<0·0001	150 (143–164)	148 (142–156)	0·0071
Diastolic blood pressure (mm Hg)	99 (91–105)	96 (90–100)	<0·0001	94 (90–100)	94 (90–99)	0·53
Oxygen saturation less than 93%	43 (3·9%)	28 (0·4%)	<0·0001	Not collected	Not collected	NA
Dipstick proteinuria (number of pluses)	2 (1–3)	1 (1–2)	<0·0001	1 (1–1)	1 (0–1)	<0·0001
**Laboratory tests—worst values on day of first assessment**						
Haematocrit (%)	0·36 (0·34–0·38)	0·36 (0·34–0·38)	0·0019	0·35 (0·32–0·38)	0·36 (0·33–0·38)	0·0683
Total leucocyte count (×109 per L)	10·9 (9·5–12·3)	10·6 (9·3–12·0)	<0·0002	9·66 (7·62–13·57)	9·9 (8·2–12)	0·73
Platelet count (×109 per L)	198 (157–235)	212 (175–245)	<0·0001	189 (126–237)	219 (182–265)	<0·0001
Mean platelet volume (fL)	9·6 (8·9–10·8)	10·0 (9·1–11·2)	<0·0001	9·5 (8·7–10·7)	9·8 (8·9–10·9)	0·28
Fibrinogen (g/L)	27·1 (25·1–29·3)	26·2 (24·6–27·9)	<0·0001	6·2 (5·4–7·4)	6·5 (5·7–7·2)	0·19
Activated partial thromboplastin time (seconds)	27·5 (25·5–30·0)	26·6 (24·8–28·2)	<0·0001	26·2 (24·37–28·85)	26·4 (24·35–27·8)	0·69
Serum creatinine (μmol/L)	64 (54–75)	60 (52–70)	<0·0001	61 (50–74·5)	54 (47–63)	<0·0001
Uric acid (mmol/L)	365 (321–418)	339 (297–385)	<0·0001	350 (297–435)	339·5 (281·25–396)	0·0758
Aspartate transaminase (U/L)	39 (29–65)	29 (22–41)	<0·0001	28 (22·5–49·5)	23 (18–31)	<0·0001
Alanine transaminase (U/L)	32 (20–55)	23 (14–35)	<0·0001	17 (11–33·25)	14 (10–20)	0·0183
Albumin (g/L)	27 (18–30)	29 (23–32)	<0·0001	34 (31–36)	35 (33–37)	<0·0001
Corticosteroid received	377 (34·8%)	1695 (21·8%)	<0·0001	Not collected	Not collected	..
Antihypertensive medication received	714 (65·9%)	4097 (52·7%)	<0·0001	53 (43·8%)	849 (30·6%)	0·0025
Magnesium sulphate received	556 (51·3%)	2257 (29·0%)	<0·0001	4 (3·3%)	15 (0·5%)	0·0069
**Pregnancy outcomes**						
Gestational age at delivery (weeks)	35·0 (31·5–37·6)	37·0 (34·1–38·7)	<0·0001	36·4 (32·9–39·4)	39 (37·3–40·1)	<0·0001
Birthweight (g)	1900 (965–2750)	2560 (1556–3200)	<0·0001	Not collected	Not collected	..
Intrauterine fetal death, ≥22^+^0 weeks or ≥500 grams^22^	65 (6·0%)	163 (2·1%)	<0·0001	6 (4·96%)	18 (0·65%)	<0·0003
Neonatal death within 28 days	40 (3·7%)	102 (1·3%)	<0·0001	2 (1·65%)	1 (0·04%)	0·0050

Data are n (%) or median (IQR). Race has been defined in the appendix (pp 4–5). NA=not applicable.

* χ^2^, Fisher's exact, or Mann-Whitney *U* test.

At presentation with pre-eclampsia, women who subsequently developed an adverse maternal outcome (*vs* those who did not) differed in their symptom profile, signs, and laboratory results, although results largely overlapped (table 1). Compared with women who did not experience outcomes, women who did experience outcomes were more often symptomatic, had lower weight, higher blood pressure, lower oxygen saturation, higher dipstick proteinuria, and more perturbed laboratory results; differences in laboratory results were not changed significantly by imputation. Women who subsequently developed an adverse outcome more often received antenatal corticosteroids, antihypertensives, and magnesium sulphate. Compared with the babies of women who did not experience outcomes, babies of women who did were born earlier and of lower birthweight, and more often died.

Table 2 shows that 590 (6·7%) of 8843 women had an adverse maternal outcome within 2 days of first assessment, 813 (9·2%) within 7 days of assessment, and 1083 (12·2%) at any time before primary discharge. Most adverse outcomes were cardiorespiratory, haematological, hepatic, or placental. There were two maternal deaths, neither within 48 h of first assessment. In the external validation group, 83 (2·9%) of 2901 women had an adverse maternal outcome within 2 days, 99 (3·4%) within 7 days, and 121 (4·2%) at any time before primary discharge. Most adverse outcomes were central nervous system, haematological, or placental.

**Table 2:** Occurrence of adverse maternal outcomes following first assessment, by mortality or morbidity event

		**PIERS-ML group (N=8843)**			**External validation group (N=2901)**		
		Within 2 days of first assessment (n=814 outcomes of 590 women)	Within 7 days of assessment (n=1156 outcomes of 813 women)	At any time before primary discharge (n=1530 outcomes of 1083 women)	Within 2 days of first assessment (n=96 outcomes of 83 women)	Within 7 days of assessment (n=115 outcomes of 99 women)	At any time before primary discharge (N=121)
Maternal death		0	1 (0·1%)	2 (0·1%)	1 (1%)	1 (0·9%)	1 (0·7%)
Central nervous system							
	Eclamptic seizures	49 (6%)	63 (5·4%)	75 (4·9%)	22 (22·9%)	22 (19·1%)	25 (17·7%)
	Glasgow coma score <13	14 (1·7%)	15 (1·3%)	20 (1·3%)	..	..	..
	Stroke or reversible ischaemic neurological deficit	4 (0·5%)	5 (0·4%)	6 (0·4%)	0	0	0
	Transient ischaemic attack	0	1 (0·1%)	1 (0·1%)	..	..	..
	Cortical blindness	3 (0·4%)	5 (0·4%)	6 (0·4%)	1 (1%)	1 (0·9%)	1 (0·7%)
	Posterior reversible encephalopathy	4 (0·5%)	5 (0·4%)	5 (0·3%)			
Cardiorespiratory							
	Positive inotropic support required	4 (0·5%)	5 (0·4%)	8 (0·5%)	..	..	..
	Infusion of a third injectable antihypertensive	17 (2·1%)	28 (2·4%)	33 (2·2%)	..	..	..
	Myocardial ischaemia or infarction	4 (0·5%)	5 (0·4%)	6 (0·4%)	2 (2·1%)	2 (1·7%)	2 (1·4%)
	Oxygen saturation less than 90%	53 (6·5%)	85 (7·4%)	114 (7·5%)	2 (2·1%)*	2 (1·7%)*	3 (2·1%)*
	At least 50% fractional inspired oxygen for at least 1 h	29 (3·6%)	50 (4·3%)	72 (4·7%)	2 (2·1%)*	2 (1·7%)*	3 (2·1%)*
	Intubation other than for caesarean birth	23 (2·8%)	34 (2·9%)	47 (3·1%)	2 (2·1%)*	2 (1·7%)*	3 (2·1%)*
	Pulmonary oedema	55 (6·8%)	82 (7·1%)	96 (6·3%)	2 (2·1%)	3 (2·6%)	3 (2·1%)
Haematological							
	Blood transfusion	243 (29·9%)	348 (30·1%)	460 (30·1%)	29 (30·2%)	36 (31·3%)	53 (37·6%)
	Platelet count <50 × 109 per L, without transfusion	85 (10·4%)	104 (9%)	112 (7·3%)	5 (5·2%)	8 (7%)	9 (6·4%)
Hepatic							
	Dysfunction	23 (2·8%)	30 (2·6%)	44 (2·9%)	5 (5·2%)	5 (4·3%)	5 (3·5%)
	Haematoma or rupture	0	0	0	2 (2·1%)	2 (1·7%)	2 (1·4%)
Renal							
	Acute renal insufficiency in women without chronic kidney disease	3 (0·4%)	5 (0·4%)	9 (0·6%)	8 (8·3%)†	8 (7%)†	8 (5·7%)†
	Acute renal failure in women with chronic kidney disease	36 (4·4%)	45 (3·9%)	52 (3·4%)	8 (8·3%)†	8 (7%)†	8 (5·7%)†
	Dialysis	2 (0·2%)	7 (0·6%)	11 (0·7%)	0	0	0
Other							
	Placental abruption	75 (9·2%)	98 (8·5%)	129 (8·4%)	17 (17·7%)	25 (21·7%)	29 (20·6%)
	Severe ascites	30 (3·7%)	50 (4·3%)	65 (4·2%)	0	0	0
	Bell's palsy	3 (0·4%)	3 (0·3%)	6 (0·4%)	0	0	0

* Respiratory failure (pulmonary oedema accompanied by severe hypoxaemia with need for intubation or mechanical ventilation).

† Acute kidney injury (serum creatinine >2 mg/dL [>176·8 mM]). All other outcomes have been defined in the appendix (pp 4–5).

Data from a random selection of 6633 women were used for model development. The following variables were excluded due to at least 60% missingness: total bilirubin, urinary protein to creatinine ratio, international normalised ratio, and lactate dehydrogenase (appendix pp 8–9). The remaining variables either contained no missingness or fewer than 60% missing at random or missing completely at random observations.

**Figure 1:**
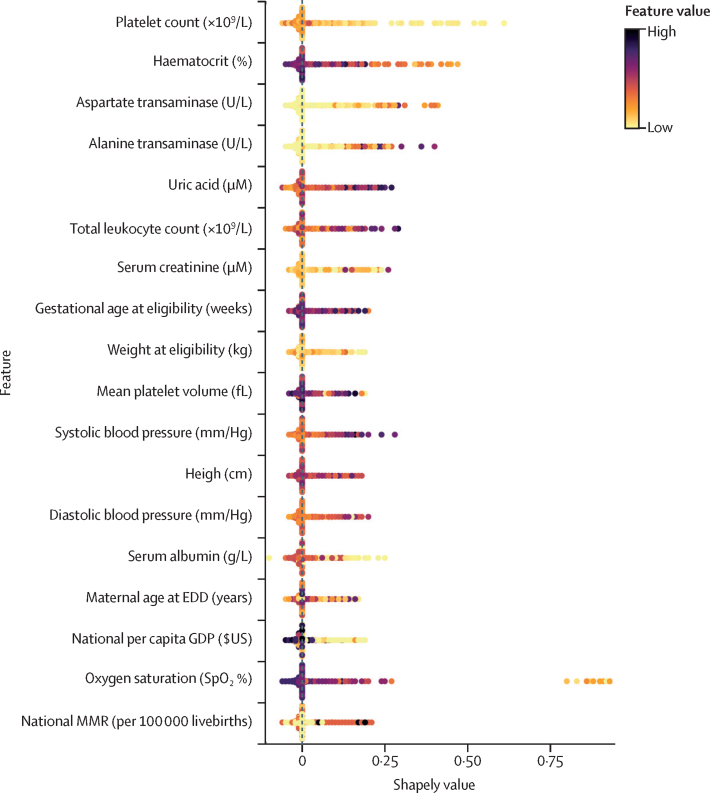
Shapley values for the PIERS-ML variables in a single random forest on the corresponding development dataset Shapley values represent the feature value's contribution to the individual's predicted probability. Positive Shapley values increase the predicted probability, negative values decrease the predicted probability, and values of zero show no change to the predicted probability. EDD=expected date of delivery. GDP=gross domestic product. SpO_2_=oxygen saturation by pulse oximetry.

**Figure 2:**
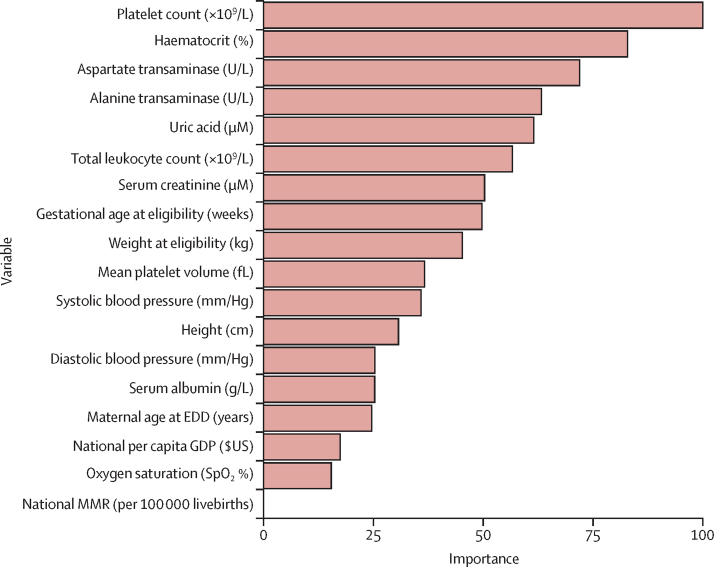
PIERS-ML variables ranked by importance within the random forest model based on Gini index, compared with the least important variable (National MMR) Random Forests enable examination of feature importances, which is the mean of the amount the Gini Index (or node impurity) decreases by in each tree at the split that uses the feature. The more the Gini Index decreases for a feature, the more important it is. This figure rates the features from 0–100, with 100 being the most important. EDD=expected date of delivery. GDP=gross domestic product. SpO_2_=oxygen saturation by pulse oximetry.

Figure 1 contains a plot of the Shapley values for a single random forest on its corresponding validation dataset. Although most Shapley values for all variables were close to zero, high values of serum creatinine, national maternal mortality ratio, aspartate and alanine transaminases, and low values of platelet count, oxygen saturation, national per capita GDP, and haematocrit had high Shapley values, meaning an increased predicted probability (appendix pp 21–22). Figure 2 shows the relative importance of the 18 PIERS-ML model variables. Platelet count was of greatest importance and national MMR was of least importance. All target organ systems were included, with the following numbers of covariates: cardiorespiratory (N=3), renal (N=3), hepatic (N=2), and haematological (N=4). In addition, there were variables representing health systems (N=2), demographic characteristics (N=2), and anthropometry (N=2).

**Figure 3:**
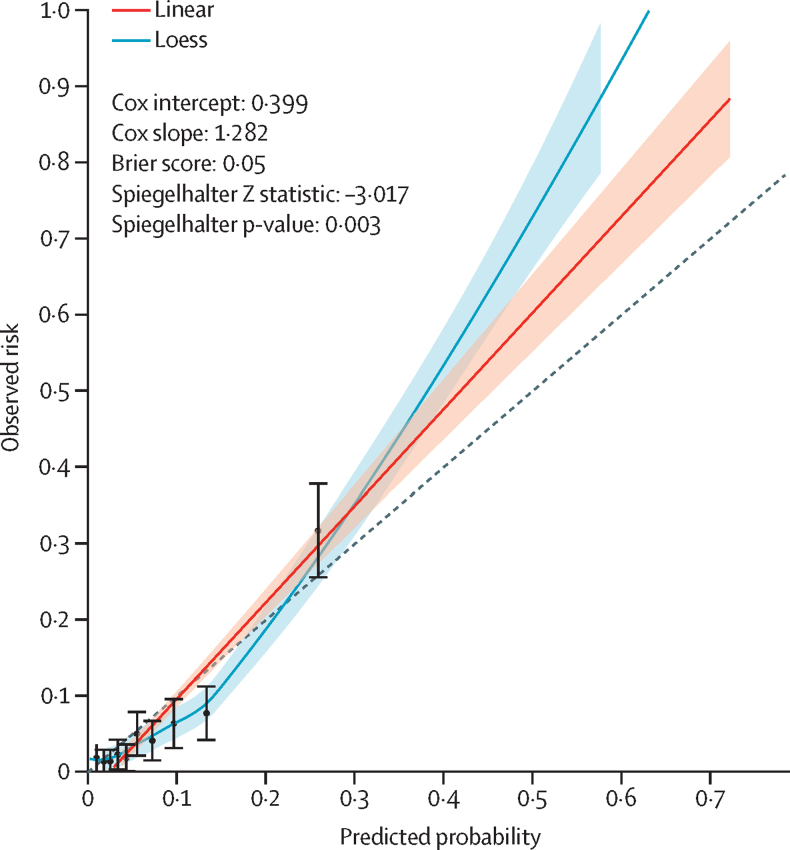
Calibration plot for the PIERS-ML model Predictions on the validation and testing datasets, in increasing order, were binned together into ten groups of 221 predictions. Event rates (observed risk) were calculated along with confidence intervals and plotted against the mean predicted probability per group to create the dot and whisker plot. Smooth lines were plotted using the individual predicted probabilities and yes and no outcomes, with Linear (red) and Loess (blue) methods. Cox calibration intercept and slope, Brier score, and Spiegelhalter Z scores were calculated.

**Figure 4:**
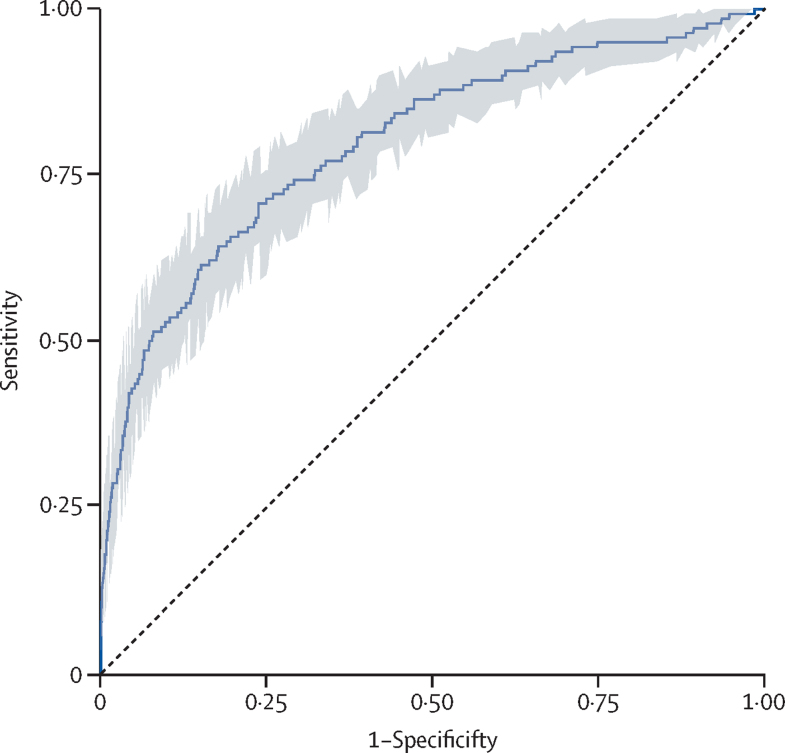
PIERS-ML area under receiver-operator characteristic for adverse maternal outcomes within 2 days of initial assessment using data within 1 day of initial assessment and before the occurrence of any outcome Area-under-the receiver-operator characteristic of 0·78 (95% CI 0·73–0·82).

**Figure 5:**
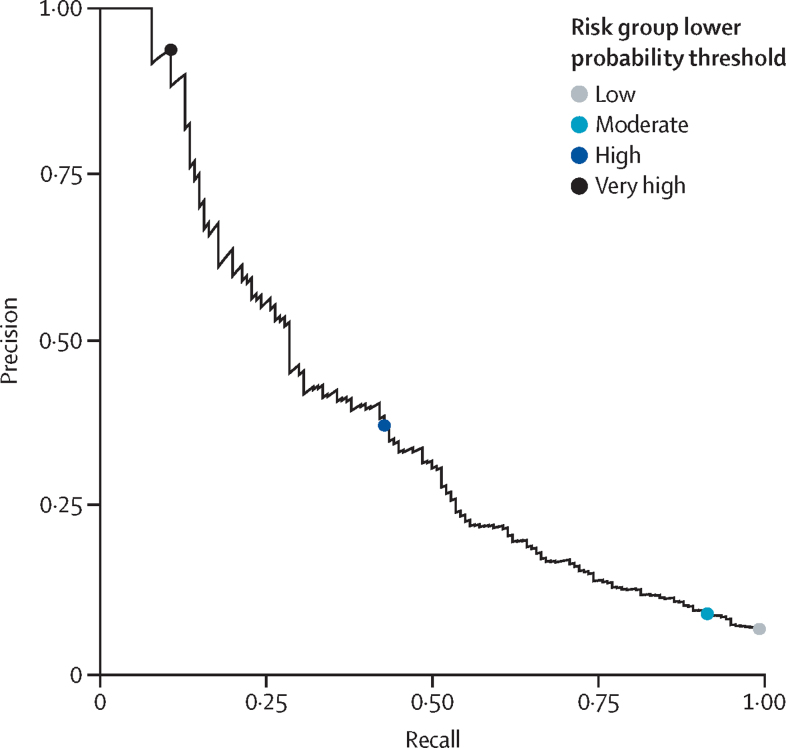
Precision-recall plot for the PIERS-ML model

From the remaining 2210 (24·9%) of 8843 women, 1107 women informed selection of cutoff points for the risk strata according to likelihood ratios, and the remaining 1103 women informed PIERS-ML model validation according to the selected cutoff points. Calibration was assessed on all 2210 women in the internal validation group; although the linear calibration curve appeared to be close to an intercept of 0 and slope of 1, the Spiegelhalter p value of 0·0026 showed the model to not be optimally calibrated (figure 3). As calibration did not improve upon recalibration (appendix pp 22–23), we chose to prioritise stratification into risk classification groups to inform clinical decision making. The PIERS-ML model accurately stratified risk for adverse maternal outcomes within 2 days (table 3), with an AUROC of 0·80 (95% CI 0·76–0·84 *vs* the currently used logistic regression model, fullPIERS: AUROC 0·68 [95% CI 0·63–0·74]; figure 4)**.** The precision-recall curve of the PIERS-ML model on the validation data was very close to a straight line, meaning that there is no single probability threshold with both good precision and good recall; therefore, using a single cutoff point to classify patients into an outcome group and a no outcome group would be inaccurate (figure 5)**.**

**Table 3:** Diagnostic test performance of PIERS-ML risk strata

	**PIERS-ML output (for outcomes within 2 days)**	**Hypertensive pregnant women in stratum**	**Hypertensive pregnant women with an outcome within 2 days**	**Hypertensive pregnant women within risk stratum with an outcome within 7 days**	**Hypertensive pregnant women within risk stratum with an outcome at any time**
**Primary combined adverse maternal outcome (AUROC 0·80 [0·76–0·84], AUPRC 0·38)**					
Very low risk	≤0·5%	8 (0·7%)	0	1 (12·5%)	1 (12·5%)
Low risk	0·6–3·0%	321 (29·1%)	7 (2·2%)	13 (4·0%)	21 (6·5%)
Moderate risk	3·1–18·6%	676 (61·3%)	36 (5·3%)	58 (8·6%)	76 (11·2%)
High risk	18·7–45·5%	87 (7·9%)	23 (26·4%)	25 (28·7%)	28 (32·2%)
Very high risk	≥45·6%	11 (1·0%)	10 (90·9%)	10 (90·9%)	10 (90·9%)
**Excluding mean platelet volume (AUROC 0·80 [0·76–0·84], AUPRC 0·39)**					
Very low risk	≤0·5%	8 (0·7%)	0	1 (12·5%)	1 (12·5%)
Low risk	0·6–3·0%	340 (30·8%)	8 (2·4%)	14 (4·1%)	22 (6·5%)
Moderate risk	3·1–18·6%	656 (59·5%)	35 (5·3%)	56 (8·5%)	74 (11·3%)
High risk	18·7–45·5%	86 (7·8%)	22 (25·6%)	25 (29·1%)	28 (32·6%)
Very high risk	≥45·6%	13 (1·2%)	11 (84·6%)	11 (84·6%)	11 (84·6%)
**Including fibrinogen and activated prothrombin time (AUROC 0·80 [0·76–0·84], AUPRC 0·38)**					
Very low risk	≤0·5%	7 (0·6%)	0	1 (14·3%)	1 (14·3%)
Low risk	0·6–3·0%	291 (26·4%)	6 (2·1%)	12 (4·1%)	20 (6·9%)
Moderate risk	3·1–18·6%	709 (64·3%)	39 (5·5%)	61 (8·6%)	79 (11·1%)
High risk	18·7–45·5%	82 (7·4%)	19 (23·2%)	21 (25·6%)	24 (29·3%)
Very high risk	≥45·6%	14 (1·3%)	12 (85·7%)	12 (85·7%)	12 (85·7%)
**fullPIERS (AUROC 0·62 [0·56–0·68], AUPRC 0·33)**					
Very low risk	≤1·3%	6 (0·5)	2 (33·3%)	2 (33·3%)	2 (33·3%)
Low risk	NA	NA	NA	NA	NA
Moderate risk	1·4–30·4%	1011 (91·5%)	51 (5·0%)	78 (7·7%)	105 (10·74)
High risk	30·5–70·6%	62 (5·6%)	11 (17·7%)	14 (22·6%)	14 (22·6%)
Very high risk	≥70·7%	26 (2·4%)	12 (46·2%)	13 (46·2%)	15 (57·7%)
**fullPIERS using PIERS-ML thresholds (AUROC 0·62 [0·56–0·68], AUPRC 0·33)**					
Very low risk	≤0·5%	3 (0·3%)	0	0	0
Low risk	0·6–3·0%	31 (2·8%)	4 (12·9%)	5 (16·1%)	5 (16·1%)
Moderate risk	3·1–18·6%	868 (79·0%)	42 (4·8%)	66 (7·6%)	89 (10·3%)
High risk	18·7–45·5%	146 (13·3%)	12 (8·2%)	16 (11·0%)	19 (13·0%)
Very high risk	≥45·6%	51 (4·6%)	18 (35·3%)	20 (39·2%)	22 (43·1%)
**fullPIERS refitted on combined dataset (AUROC 0·67 [0·62–0·72], AUPRC 0·25)**					
Very low risk	≤1·0%	1 (0·1%)	0	0	0
Low risk	NA	NA	NA	NA	NA
Moderate risk	1·1–15·5%	1031 (93·3%)	60 (5·8%)	87 (8·4%)	115 (11·2%)
High risk	15·6–44·2%	64 (5·8%)	11 (17·2%)	15 (23·4%)	16 (25·0%)
Very high risk	≥44·3%	9 (0·8%)	5 (55·6%)	5 (55·6%)	5 (55·6%)
**fullPIERS refitted on combined dataset using PIERS-ML thresholds (AUROC 0·67 [0·62–0·72], AUPRC 0·25)**					
Very low risk	≤0·5%	0	NA	NA	NA
Low risk	0·6–3·0%	108 (9·8%)	5 (4·6%)	6 (5·6%)	13 (12·0%)
Moderate risk	3·1–18·6%	943 (85·8%)	57 (6·0%)	85 (9·0%)	105 (11·1%)
High risk	18·7–45·5%	40 (3·6%)	9 (22·5%)	11 (27·5%)	12 (30·0%)
Very high risk	≥45·6%	8 (0·7%)	5 (62·5%)	5 (62·5%)	5 (62·5%)
**External validation cohort (AUROC 0·76 [0·71–0·82], AUPRC 0·17)**					
Very low risk	≤0·5%	9 (0·3%)	0	0	0
Low risk	0·6–3·0%	1512 (52·1%)	17 (1·1)	22 (1·5)	34 (2·2)
Moderate risk	3·1–18·6%	1324 (45·7%)	47 (3·5)	55 (4·2)	65 (4·9)
High risk	18·7–45·5%	52 (1·8%)	17 (32·7)	20 (38·5)	20 (38·5)
Very high risk	≥45·6%	3 (0·1%)	2 (66·7)	2 (66·7)	2 (66·7)

Data are n (%). Risk strata determined by diagnostic test performances for first occurrence of any component of the primary combined adverse maternal outcome within 2 days: very low risk –LR <0·1; low risk –LR 0·1 to 0·2; moderate risk +LR <5·0 and –LR >0·2; high risk +LR 5·0 to 10·0; very high risk +LR >10·0. AUROC=area under the receiver-operator characteristic. AUPRC=area under the precision-recall curve. NA=could not select a probability threshold greater than the very low risk threshold that produced a negative likelihood ratio less than or equal to 0·2, when using predicted probabilities from the fullPIERS model.

Of the 1103 women in the internal validation group, 329 (29·8%) women were classified as being at very low risk (eight [0·7%] women) or low risk (321 [29·1%] women) during the 2 days (table 3). Among women at very low risk, adverse maternal outcomes were very infrequent: zero within 2 days, and one (12·5%) of eight women each within 7 days or at any time. Among women at low risk, adverse maternal outcomes were also infrequent: seven (2·2%) of 321 women within 2 days, 13 (4·0%) women within 7 days, and 21 (6·5%) women at any time. The PIERS-ML model correctly classified women at very low and low risk of adverse outcomes within 2 days, but not within 7 days or at any time.

98 (8·9%) of 1103 women were classified as being at high (87 [7·9%] women) or very high risk (11 [1·0%] women) of an adverse maternal outcome within 2 days (table 3). Among women at high risk, adverse maternal outcomes were frequent: 23 (26·4%) of 87 women within 2 days, 25 (28·7%) women within 7 days, and 28 (32·2%) women at any time. Among women at very high risk, adverse maternal outcomes were very frequent, with all ten (90·9%) of 11 women's outcomes occuring within 2 days. The PIERS-ML model correctly classified women at high and very high risk of adverse outcomes within 2 days, 7 days, or at any time.

Among 676 (61·3%) of 1103 women classified as being at moderate risk, adverse maternal outcomes occurred in 36 (5·3%) women within 2 days, 58 (8·6%) women within 7 days, and 76 (11·2%) women at any time, all within the anticipated range of outcomes based on uninformative likelihood ratios (table 3).

The event rate in the external validation group was 2·9% (83 of 2901 women) in 2 days, 3·4% (99 women) in 7 days, and 4·2% (121 women) at any point, compared with 6·7% in 2 days, 9·2% in 7 days, and 12·2% at any point in the combined dataset. From the 2901 women in the external validation cohort, nine (0·3%) women were classified as being at very low risk, 1512 (52·1%) women were classified as being at low risk, 1324 (45·7%) women were classified as being at moderate risk, 52 (1·8%) women were classified as being at high risk, and three (0·1%) women were classified as being at very high risk. The PIERS-ML model accurately stratified risk for adverse maternal outcomes within 2 days with women assigned to each stratum experiencing an outcome rate within the predicted range, and an AUROC of 0·76 (95% CI 0·71–0·82; table 3). Although the event rates were lower in the external validation set than the development data, the model still correctly classified women at very low risk and low risk, as well as those at high risk and very high risk.

Excluding mean platelet volume from the set of variables significantly altered the PIERS-ML model's performance, resulting in 59·5% (656 of 1103 women) in the moderate risk stratum, and loss of reassurance for the very low risk and low risk strata beyond 2 days (table 3), although the AUROC remained unchanged (0·80 [0·76–0·84]). Including fibrinogen and activated prothrombin time as variables neither improved the model's performance (AUROC 0·80 [0·76–0·84]) nor its risk stratification capacity (moderate risk stratum 64·3% (709 of 1103; table 3); as there was no significant difference between model performance, the model with no coagulation variables was chosen as coagulation variables are often not available or expensive to obtain if not necessary. The original fullPIERS model had poorer performance in the combined validation dataset (table 3).

The PIERS-ML model had strong performance in ruling out (very low risk and low risk strata) and ruling in (high risk and very high risk strata) subsequent seizures of eclampsia (table 4). For the 2210 women in the full internal validation group, stillbirth risks varied by risk strata, such that women at very low risk and low risk of adverse maternal events had few stillbirths, whereas those at moderate risk had 2·8% (32 of 1148) stillbirths, high risk had 11·6% (16 of 138 women) stillbirths, and very high risk had 0·0% stillbirths (table 4).

**Table 4:** Secondary analyses for eclampsia and stillbirth

	**Hypertensive pregnant women in stratum**	**Hypertensive pregnant women within risk stratum with an outcome within 2 days**		**Hypertensive pregnant women within risk stratum with an outcome within 7 days**		**Hypertensive pregnant women within risk stratum with an outcome at any time**	
	N (%)	N (%)	Likelihood ratios	N (%)	Likelihood ratio	N (%)	Likelihood ratio
**Eclampsia (N=140 maternal events, AUROC 0·80 [95% CI 0·69–0·91])**							
Very low risk	8 (0·7%)	0	−LR 0·0 (0·0 to NaN)	0	−LR 0·0 (0·0 to NaN)	0	−LR 0·0 (0·0 to NaN)
Low risk	321 (29·1%)	0	−LR 0·0 (0·0 to NaN)	0	−LR 0·0 (0·0 to NaN)	0	−LR 0·0 (0·0 to NaN)
Moderate risk	676 (61·3%)	3 (0·4%)	..	3 (0·4%)	..	2 (0·3%)	..
High risk	87 (7·9%)	2 (2·3%)	+LR 5·1 (1·7 to 15·3)	2 (2·3%)	+LR 5·1 (1·7 to 15·3)	5 (5·4%)	+LR 8·9 (5·3 to 14·8)
Very high risk	11 (1·0%)	1 (9·1%)	+LR 18·3 (2·8 to 121·3)	1 (9·1%)	+LR 18·3 (2·8 to 121·3)	1 (7·7%)	+LR 11·5 (1·7 to 78·3)
**Stillbirth (N=50 stillbirths of 1925 women with informative data within the complete validation dataset, AUROC 0·79 [0·74–0·85])**							
Very low risk	23 (1·2%)	0	−LR 0·0 (0·0 to NaN)	0	−LR 0·0 (0·0 to NaN)	0	−LR 0·0 (0·0 to NaN)
Low risk	601 (31·2%)	1 (0·2%)	−LR 0·1 (0·0 to 0·4)	1 (0·2%)	−LR 0·1 (0·0 to 0·4)	1 (0·2%)	−LR 0·1 (0·0 to 0·4)
Moderate risk	1148 (59·6%)	32 (2·8%)	..	32 (2·8%)	..	33 (2·9%)	..
High risk	138 (7·2%)	16 (11·6%)	+LR 5·0 (3·2 to 7·7)	16 (11·6%)	+LR 5·0 (3·2 to 7·7)	16 (11·6%)	+LR 4·9 (3·1 to 7·6)
Very high risk	15 (0·8%)	0	+LR 0·0 (0·0 to NaN)	0	+LR 0·0 (0·0 to NaN)	0	+LR 0·0 (0·0 to NaN)

Risk strata determined by diagnostic test performances for first occurrence of any component of the primary combined adverse maternal outcome within 2 days: very low risk –LR <0·1; low risk –LR 0·1 to 0·2; moderate risk +LR <5·0 and –LR >0·2; high risk +LR 5·0 to 10·0; very high risk +LR >10·0. AUROC=area under the receiver-operator characteristic. Inf=infinity. –LR=negative likelihood ratio. +LR=positive likelihood ratio. NaN=not a number. Likelihood ratios are presented in addition to stratification performance as the PIERS-ML model was not developed to predict either of these two outcomes.

Removing renal and haematological components of the outcome did not significantly alter PIERS-ML performance (appendix pp 10–11). Alternative machine learning strategies were considered but did not show improved performance (appendix pp 12–14). The complete case analysis showed a reduced AUROC but with wider confidence intervals that included the AUROC of the imputed model. The validation with mean imputation had both similar number of patients per risk strata and similar number of outcomes as with imputation (appendix pp 15–16, 25).

## Discussion

This study included 8843 women from 11 countries presenting for first assessment of pre-eclampsia and used the random forest method to develop and internally validate an 18-variable model for maternal risk stratification, applicable for LMICs and high-income countries. A further 2901 UK resident women contributed data to a fully external validation dataset.

The PIERS-ML model has identified nearly 40% of women with pre-eclampsia for whom care should be altered. The 29·8% (329 of 1103 women) and their families and maternity care providers identified as being at very low risk (0·7% [eight of 1103 women]) or low risk (29·0% [321 women]) can be reassured that it is very unlikely that adverse maternal events will occur within 2 days. However, for the 8·9% (98 women) identified to be a high risk (7·9% [87 women]) or very high risk (1·0% [11 women]), a timely clinical response can be justified based on a substantial risk of an adverse maternal event within 2 days, or for women at very high risk, at any time. Identifying these women can inform discussions about place of care, transfer of care, antenatal and postnatal surveillance, co-interventions, and timed birth.

Strengths of our study include the large sample size, which was, to our knowledge, larger than any previous study modelling adverse outcomes in women with pre-eclampsia.^5^, ^8^, ^12^, ^20^, ^23^ We tested a list of variables with clinical external validity and availability, including all target organ systems, except for the central nervous system. Clinical central nervous system predictors rely on either the subjectivity of symptoms or the questionable reproducibility of deep tendon reflexes and clonus, particularly in pregnancy. Also, the PIERS-ML model does not include direct measures of coagulation; these are not routinely performed, and their addition neither altered model performance nor warranted related costs in women with pre-eclampsia. Machine learning is suitable for managing many variables, without assumption with respect to interactions and mediation, and addresses concerns regarding collinearity. Trade-offs were considered between model performance, complexity, and face validity. In addition, machine learning algorithms can use all available predictor variables as inputs as variables that are not predictive of the outcome will have little to no effect on the predicted probabilities. Therefore, a machine learning-based model with more variables than necessary should not have degraded performance in the context of this application. However, including unnecessary variables means that included variables might not contribute substantially to the predictions. To make the model more pragmatic in a clinical setting, we have chosen to use feature selection to remove unimportant variables avoiding unnecessary data collection or clinical tests.

There are significant differences between our PIERS-ML model and the recently published Charité machine learning-based model.^23^ Schmidt and colleagues^23^ used data from 1647 women with pre-eclampsia admitted to a single high-income country institution, and those data were modelled against a composite maternal and fetal outcome, despite rates of adverse maternal and perinatal outcomes not tracking together.^24^, ^25^ Although they applied different machine learning methods, they used approximately 24 observations (internally normalised to multiples of the median) per candidate variable, compared with approximately 230 observations per variable in the PIERS-ML dataset. They undertook no imputations, thereby assuming that missingness is predictive of either outcome or no outcome. In addition, we fitted models using ten-fold cross-validation to minimise overfitting and validated the model in an independent 12·5% (*vs* 10% from the Charité model) of the dataset. Importantly, our risk stratification was data driven in ruling out or ruling in the risk of adverse maternal outcomes. The main strength of the study by Schmidt and colleagues^23^ was the inclusion of angiogenic markers, whereas the main strengths of our study are the diversity of data, the use of multiple imputation, and external validation.

Missing values were common in our dataset; however, where appropriate, missingness was handled by multiple imputation, with chained random forest to minimise bias. Of note, development and validation datasets were imputed separately, thereby creating independent model development and validation datasets. Some variables (namely bilirubin, urinary protein to creatinine ratio, international normalised ratio, and lactate dehydrogenase) had to be excluded due to high levels of missingness. This limitation reflects current clinical practice. Modelling on complete data from a representative and diverse sample population will always give a more accurate result than modelling on a dataset with a substantial amount of missingness. However, obtaining such a dataset would be very difficult as many of these variables are not regularly collected in clinical practice from patients with pre-eclampsia, especially close to full term. Additionally, international normalised ratio and lactate dehydrogenase were not expected to be strong predictors, whereas urinary protein to creatinine ratio would be anticipated to outperform the readily available dipstick proteinuria.

The composite adverse maternal outcome for the PIERS model was Delphi-derived, similarly to the core maternal outcome list (iHOPE) for pre-eclampsia.^26^ However, there are differences: only PIERS includes uncontrolled hypertension, inotropic support, myocardial ischaemia or infarction, hepatic dysfunction, or transfusion, and only the iHOPE outcome set contains elevated liver enzymes, postpartum haemorrhage, and admission to intensive care. To confirm model performance, as some factors are both predictors and components of the combined outcome (eg, serum creatinine and platelet count), we assessed the PIERS-ML model excluding renal or haematological components of the outcome, or both (appendix pp 10–11).

The PIERS-ML model also proved to be highly effective on external validation in a UK resident cohort, achieving the predicted outcome rates in the risk classification strata expected from internal validation. The model could identify women for whom a timely response can be justified very well while reassuring women who were predicted very low risk who had no outcomes at any point. However, the full range of the components of the combined maternal outcome were not readily available, and some outcomes (eg, renal) were conflated within the dataset as those data had been collected previously. Although this introduces the possibility of underestimating the outcome rate as defined by the original PIERS combined outcome, we are confident in the model's ability to classify into risk strata, especially for ruling in outcomes in the high risk and very high risk strata.

Multivariable model-based risk stratification of women with pre-eclampsia is recommended by national and international clinical practice guidelines.^1^, ^27^, ^28^ With access to full laboratory facilities, models have been based on either logistic regression or a survival model for time-to-adverse event.^8^, ^20^ Women with a hypertensive disorder of pregnancy (including pre-eclampsia) in LMICs, without ready access to laboratory tests, benefit from the demographics, symptom, and signed-based miniPIERS model, with model performance improved by pulse oximetry.^5^, ^29^

The PIERS-ML model improves on previous models in several ways. First, to our knowledge, the PIERS-ML model is the first model for risk stratification in pre-eclampsia that has been developed using machine learning from women with pre-eclampsia living in LMICs (sub-Saharan Africa, South America, south Asia, and Oceania—areas of the world where more than 99% of pre-eclampsia-related maternal mortality occurs)^6^ and high-income countries. We are unaware of another model that has included data from 11 low-income, middle-income, and high-income countries.

Uniquely, the model includes national per capita gross domestic product and maternal mortality ratio, which are variables that adjust for location, avoiding adaptation of models to local outcome rates, particularly in which information governance and research resources are absent or limited. Adjusting for local settings was required for original fullPIERS model validation in LMICs.^18^ Therefore, we were not surprised by the poor performance of fullPIERS within this combined dataset. This approach to create auto-adjustment for setting should be validated in further geographies.

Second, the PIERS-ML model does not include maternal symptoms, the inclusion of which was criticised as a weakness of previous models,^8^ given the subjective nature, variable definitions, and inconsistent documentation of symptoms in health records. However, mean platelet volume, which is a marker of platelet consumption and release of immature platelet forms, is important within the PIERS-ML model.^30^ Haematology analysers routinely measure, but many laboratories do not report, mean platelet volume; our findings suggest mean platelet volume should be reported for all hypertensive pregnant women.

Third, although the PIERS-ML model does not include symptoms of central nervous system involvement, the model has clinically-relevant performance in identifying women at both least (very low risk and low risk strata) and greatest (high risk and very high risk strata) risk of developing eclampsia, especially within 7 days of initial assessment (table 4). These results could guide the targeted use of magnesium sulphate and reduce the number-needed-to-treat, for the prevention of eclampsia.^31^ Depending on health system resilience, women in the moderate risk strata might or might not be considered for magnesium sulphate prophylaxis. For women with disease onset before 34^+0^ weeks’ gestation, a loading dose of magnesium sulphate should be administered to reduce the risk of prematurity-related cerebral palsy.^32^

Fourth, women in the very low risk and low risk strata were very unlikely to suffer a stillbirth (table 4), and we believe that such women can be appropriately reassured by our model. All intrauterine fetal deaths were noted within 2 days of admission with pre-eclampsia. However, it was notable that the women in the moderate risk and high risk strata bore the greatest risk of stillbirth, presumably as maternity care providers were sufficiently concerned by the condition of women in the very high risk stratum to intervene for either maternal or fetal indications, or both, or in response to the woman experiencing an adverse maternal event. In addition, the very high risk stratum had a small sample size; therefore, performance in predicting stillbirth could not be accurately assessed.

Finally, the PIERS-ML model has been externally validated with very good performance in an independent cohort of women with pre-eclampsia admitted to hospitals that did not participate in the fullPIERS development and validation projects, and where neither fullPIERS nor miniPIERS were used in routine clinical practice.

There are three important lines of investigation that follow from our work. The first is that use of this method offers the potential for the accuracy of the model to improve over time as data accumulate and the model learns with regularly scheduled manual updates provided to model users. Amassing such data is feasible, as all individual-level variables in the PIERS-ML model are part of routine clinical and laboratory assessment of women with pre-eclampsia in well-resourced settings, and available from electronic health records in real-time. The second line of investigation is to explore whether addition of new markers could improve the PIERS-ML model performance. As markers of uteroplacental dysfunction of pre-eclampsia, angiogenic markers are used increasingly in the investigation of women with suspected pre-eclampsia, at first presentation, for ongoing surveillance^33^ (although performance in trials has been variable^34^, ^35^), and within the Charité model.^23^ If independently informative, angiogenic markers could be incorporated into the model during clinical implementation. An ophthalmic artery Doppler could provide useful information about the less-accessible intracranial circulation.^36^

Third, to determine how best to assess evolving risk among women with pre-eclampsia, especially those women at moderate risk who will require ongoing, close surveillance, particularly during the first 7 days when most adverse outcomes occur. PIERS-ML, and other models, perform best during the first 2–7 days; women with pre-eclampsia could be expectantly managed for up to 4 weeks.^2^ Although repeated risk stratification has been recommended,^27^ future work should examine replacing this serial static model approach with a new dynamic approach that accounts for changes over time.

The PIERS-ML model uses the power of machine learning to develop a new effective risk stratification tool for international use. Using only variables routinely assessed in pregnant women with pre-eclampsia, this tool can stratify women with the condition into clinically-relevant risk strata, to guide clinical care, and is amenable to future inclusion of new biomarkers and predictors.

## Data sharing

The PIERS data are de-identified participant-level data. As permitted by existing data sharing and collaboration agreements, the data will be available to academically active entities (eg, universities, non-governmental organisations, and multilaterals), with the PIERS Principal Investigator (Peter von Dadelszen), or named delegate, as a named co-investigator, for the purposes of pregnancy hypertension-related research and within the limits of the informed consent obtained. Access will be through the Principal Investigator, or named delegate, contacted at pvd@kcl.ac.uk. A full data dictionary and all study documents will be available. Access will be through written application. The complete data sharing statement for the PIERS-ML model is in the appendix (p 11).

## Declaration of interests

TM-C, KK, PM, SJEB, LAM, and PvD acknowledge that the intellectual property related to the PIERS-ML model has been registered, and that the inventors have no financial benefit from the use of the model based on the transfer. TM-C was funded by the University of Strathclyde, through the STRADDLE (University of Strathclyde Diversity in Data Linkage) Centre for Doctoral Training. All other authors declare no competing interests.
