# Supplementary material for: Machine learning-enabled maternal risk assessment for women with pre-eclampsia (the PIERS-ML model): a modelling study
Source: Lancet Digit Health. 2024 Mar 20;6(4):e238–50. doi: 10.1016/S2589-7500(23)00267-4 (PMC10983826; doi:10.1016/S2589-7500(23)00267-4)
Supplement: Supplementary appendix [file mmc1.pdf]

THE LANCET
Digital Health

Supplementary appendix

This appendix formed part of the original submission and has been peer reviewed. We post it as supplied by the authors.

Supplement to: Montgomery-Csobán T, Kavanagh K, Murray P, et al. Machine learning-enabled maternal risk assessment for women with pre-eclampsia (the PIERS-ML model): a modelling study. *Lancet Digit Health* 2024; **6:** e238–50.

Supplementary Appendix

Table S1: PIERS Consortium and participating hospitals **pp 2-4**

Table S2: Definitions of Race and Adverse Maternal Outcomes **pp 5-6**

Table S3: Development and Internal Validation Cohort Characteristics **pp 7-8**

Table S4: Breakdown of Missing Data **pp 9-10**

Table S5: Sensitivity Analyses of Model Performance Excluding Organ Systems **pp 11-12**

Table S6: PIERS-ML Model Performance Using Alternative Machine Learning Strategies **pp 13-14**

Table S7: Complete case analysis of the PIERS-ML and fullPIERS models **p 15**

PIERS-ML Data Sharing Statement **p 16**

PIERS-ML Statistical Supplement **pp 17-25**

Table S1 PIERS Consortium

Country and Primary Affiliation / Participating Hospitals	Given Name	Family Name (*Delphi consensus)	
**Australia**			
University of New South Wales, Sydney, NSW	Mark A.	Brown	*
	Gregory K.	Davis	*
University of Western Australia, Crawley / King Edward Memorial Hospital for Women, Osborne Park Hospital, Osborne Park, WA	Claire	Parker	
	Barry N.	Walters	*
**Brazil**			
Universidade Federal de São Paulo / Maternidade de Vila Nova Cachoeirinha, São Paulo	Nelson	Sass *	
**Canada**			
University of British Columbia, Vancouver, BC / BC Women's Hospital and Health Centre, St Paul's Hospital, Vancouver, BC; the Richmond Hospital, Richmond, BC; Surrey Memorial Hospital, Surrey, BC	J. Mark	Ansermino	*
	Vivien	Cao	
	Geoffrey W.	Cundiff	
	Emma C.M.	von Dadelszen	
	M. Joanne	Douglas	*
	Guy A.	Dumont	
	Dustin T.	Dunsmuir	
	Jennifer A.	Hutcheon	
	K.S.	Joseph	
	Sayrin	Lalji	
	Tang	Lee	
	Jing	Li	
	Kenneth I.	Lim	*
	Sarka	Lisonkova	
	Paula	Lott	
	Jennifer M.	Menzies	
	Alexandra L.	Millman	
	Lynne	Palmer	
	Beth A.	Payne	
	Ziguang	Qu	
	James A.	Russell	*
	Diane	Sawchuck	
	Dorothy	Shaw	
	D. Keith	Still	
	U. Vivian	Ukah	
	Brenda	Wagner	
	Keith R.	Walley	*
East Kootenay Regional Hospital, Cranbrook, BC	Dany	Hugo	
University of Ottawa / the Ottawa Hospital, Ottawa, ON	The late Andrée	Gruslin	*
	George	Tawagi	
Queen's University / Kingston General Hospital, Kingston, ON	Graeme N.	Smith	*
Université de Sherbrooke / centre hospitalier universitaire de Sherbrooke, Sherbrooke, QC	Anne-Marie	Côté	*
	Jean-Marie	Moutquin	*
	Annie B.	Ouellet	
University of Toronto, Toronto, ON	Shoo K.	Lee	*
**P.R. China**			
Tongji University, Shanghai	Tao	Duan	*
	Jian	Zhou	*

**Fiji**			
University of the South Pacific / Colonial War Memorial Hospital, Suva	The late Farizah	Haniff	
	Swati	Mahajan	*
	Amanda	Noovao	*
**Finland**			
University of Helsinki, Helsinki (FINNPEC consortium) / Helsinki University Hospital, Helsinki; Kuopio University Hospital, Kuopio; Oulu University Hospital, Oulo; Tampere University Hospital, Tampere; Turku University Central Hospital, Turku	Hanna	Karjalainend	
	Alja	Kortelainen	
	Hannele	Laivuori	
**The Netherlands**			
Amsterdam University Medical Centre, Amsterdam	J. Wessel	Ganzevoort	
University of Groningen, Groningen	Henk	Groen	
**New Zealand**			
University of Otago / Christchurch Women's Hospital, Christchurch	Phillipa M.	Kyle	*
	M. Peter	Moore	*
	Barbra	Pullar	
**Pakistan**			
Aga Khan University, Karachi / Aga Khan University Hospital, Garden Hospital, Karimabad, Hospital, Kharadar Hospital, Karachi; Aga Khan Maternity & Child Care Centre, Liaqat University of Medical Sciences, Hyderabad, Sindh	Zulfiqar A.	Bhutta	*
	Rahat N.	Qureshi	
	Rozina	Sikandar	
Jinnah Post-graduate Medical College & Centre, Karachi, Sindh	The late Shereen Z.	Bhutta	*
**South Africa**			
Stellenbosch University, Stellenbosch / Tygerberg Hospital, Cape Town, Western Cape	Garth	Cloete	
	David R.	Hall	*
	The late Erika	van Papendorp	
	D. Wilhelm	Steyn	*
**Uganda**			
Makerere University / Mulago Hospital, Kampala	Christine	Biryabarema	
	Florence	Mirembe	
	Annettee	Nakimuli	
**United Kingdom**			
University of Birmingham, Birmingham (PREP Study Group) / Birmingham Women's Hospital, City Hospital, Birmingham; Wansbeck General Hospital, Ashington; North Devon District Hospital, Barnstaple; Furness General Hospital, Barrow-in-Furness; Basingstoke & North Hampshire Hospital, Basingstoke; Blackpool Victoria Hospital, Blackpool; Pilgrim Hospital, Boston; Bradford Royal Infirmary, Bradford; Queen's Hospital, Burton-on-Trent; University Hospital of Wales, Cardiff; Cumberland Infirmary, Carlisle; Countess of Chester Hospital, Chester; Chesterfield & North Derbyshire Royal Hospital, Chesterfield; University Hospitals Coventry & Warwickshire, Coventry; Leighton Hospital, Crewe; Russells Hall Hospital, Dudley; Queen Elizabeth Hospital, Gateshead; Leicester Royal Infirmary, Leicester; Lincoln County Hospital, Lincoln; Liverpool Women's Hospital, Liverpool; Barts Health NHS Trust, Royal Free Hospital, St George's Hospital, North Middlesex Hospital, West Middlesex University Hospital, London; James Cook University Hospital, Middlesbrough; Royal Victoria Infirmary, Newcastle; Derriford Hospital, Plymouth; Queen Alexandra Hospital, Portsmouth; Rotherham Hospital, Rotherham; Glan Clwyd Hospital, Rhyl; Royal Shrewsbury Hospital, Shrewsbury; Wexham Park Hospital, Slough; Southend Hospital, Southend; Staffordshire General Hospital, Stafford; City General Hospital, Stoke-on-Trent;	John	Allotey	
	Shakila	Thangaratinam	

Sunderland Royal Hospital, Sunderland; King's Mill Hospital, Sutton-In-Ashfield; Singleton Hospital, Swansea; Royal Cornwall Hospital, Truro; Warrington Hospital, Warrington; Warwick Hospital, Warwick; West Cumberland Hospital, Whitehaven; New Cross Hospital, Wolverhampton; Worcestershire Royal Hospital, Worcester; York Hospital, York			
Fetal Medicine Foundation / King's College Hospital (Demark Hill & Princess Royal University Hospital campuses), London	Kypros H.	Nicolaides	
	Olivia	Ionescu	
	Argyro	Syngelaki	
Imperial College London, London	Michael	de Swiet	*
King's College London, London	Laura A.	Magee	*
	Peter	von Dadelszen	*
Medway Maritime Hospital, Gillingham	Ranjit	Akolekar	
University of Leeds / St James' Hospital, Leeds General Infirmary, Leeds	James J.	Walker	*
University of Newcastle, Newcastle	Stephen C.	Robson	*
University of Nottingham / Nottingham City Hospital, Queen's Medical Centre, Nottingham	Fiona	Broughton-Pipkin	*
	Pamela	Loughna	*
University of Oxford / John Radcliffe Hospital, Oxford	Manu	Vatish	
	Christopher W.G.	Redman	*
University of Strathclyde, Glasgow	Sarah J.E.	Barry	
	Kimberley	Kavanagh	
	Tunde	Montgomery-Csobán	
	Paul	Murray	
	Chris	Robertson	
**United States**			
Preeclampsia Foundation, Melbourne, FL	Eleni Z.	Tsigas	
University of California San Diego, La Jolla, CA (PETRA Study Group) / Women's Healthcare, West Coast OB/GYN, San Diego, Medical Group, San Jose, CA; Baptist Health Lexington, Lexington, Norton Healthcare, Louisville, KY; University of Mississippi, Jackson, MS; University of North Carolina, Chapel Hill, Carolinas HealthCare System, Charlotte, NC; Phoenix OB/GYN, Moorestown, Saint Peter's University Hospital, New Brunswick, NJ; Obstetrix Columbia University Medical Center, New York, NY; Greater Cincinnati OB/GYN Inc, Cincinnati, ProMedica Physician Group, Toledo, OH; Corvallis Clinic, Corvallis, Oregon Health & Science University, Portland, OR; Medical University of South Carolina, Charleston, SC; Regional Obstetrical Consultants, Chattanooga, TN; Maternal Fetal Services of Utah, Salt Lake City, UT	Douglas A.	Woelkers	
University of Chicago, Chicago, IL	Marshall D.	Lindheimer	*
The University of Ohio, Columbus, OH	William A.	Grobman	*
University of Texas Health Science Center at Houston, Houston, TX	Baha M.	Sibai	
**World Health Organization**			
UNDP/UNFPA/WHO/World Bank Special Programme of Research, Development and Training in Human Reproduction (HRP), Department of Reproductive Health and Research (RHR), Geneva, Switzerland	Mario	Merialdi	
	Mariana	Widmer	

* Member of the PIERS outcome Delphi consensus

Table S2 Definitions of Race and Adverse Maternal Outcomes

Outcome	Definition
Race	
White	Origins in Europe, Middle East, North Africa [Arabic origins], Western Russia [including Afghanistan and South Russia] and Hispanics of European origin
Asian	Origins in the Indian sub-continent [e.g., India, Pakistan, Bangladesh, and Sri Lanka], or in the Far East and Southeast Asia [e.g., China, Japan, Korea, Philippines, Thailand, Eastern Russia]
Black	Origins in any of the original peoples of Africa
Other	Including mixed ancestry
Mortality	Maternal death occurring within six weeks of pregnancy or if later, attributable to complications of preeclampsia
Hepatic dysfunction	International normalised ratio (INR) >1.2 in the absence if disseminated intravascular coagulation (DIC) or treatment of warfarin (DIC is defined as having both: abnormal bleeding and consumptive coagulopathy [i.e., low platelets, abnormal peripheral blood film, or one or more of the following: increased INR, increased prothrombin time (PTT), low fibrinogen, of increased fibrin degradation products that are outside normal non-pregnancy ranges])
Hepatic hematoma or rupture	Blood collection under the hepatic capsule as confirmed by ultrasound or laparotomy
Glasgow coma score (GCS) <13	Based on GCS scoring system: Teasdale G, Jennet B. Assessment of coma and impaired consciousness: a practical scale. *Lancet* 1974; **2**:81-83
Stroke	Acute neurological event with deficits lasting longer than 48 hours
Cortical blindness	Loss of visual acuity in the presence of intact papillary response to light
Reversible Ischaemic Neurologic Deficit (RIND)	Cerebral ischaemia lasting longer than 24 hrs but less than 48 hours revealed through clinical examination
Retinal detachment	Separation of the inner layers of the retina from the underlying retinal pigment epithelium (RPE, choroid) and is diagnosed by ophthalmological exam
Acute renal insufficiency	For women with no underlying renal disease, defined as serum creatinine >150 µM
Acute renal failure	For women with an underlying history of renal disease, defined as serum creatinine >200 µM
Dialysis	Including haemodialysis and peritoneal dialysis

Postpartum haemorrhage (PPH) requiring transfusion or hysterectomy	Occurrence of PPH that required transfusion or hysterectomy
Placental abruption	Any occurrence of abruption diagnosed clinically or based on placental pathology report
Platelet count < 50,000 x 10^9/L without blood transfusion	Measurement of platelet count recorded as less than 50,000 x 10^9/L without patient receiving a blood transfusion
Transfusion of blood products	Includes transfusion of any units of blood products: fresh frozen plasma (FFP), platelets, red blood cells (RBCs), cryoprecipitate (cryo) or whole blood
Positive inotropic support	The use of vasopressors to maintain a systolic blood pressure >90 mmHg or mean arterial pressure >70 mmHg
Myocardial ischaemia/infarction	Electrocardiogram (ECG) changes (ST segment elevation or depression) without enzyme changes AND/OR any one of the following: 1) Development of new pathologic Q waves on serial ECGs. The patient may or may not remember previous symptoms. Biochemical markers of myocardial necrosis may have normalised, depending on the length of time that has passed since the infarct developed. 2) Pathological findings of an acute, healed or healing MI 3) Typical rise and gradual fall (troponin) or more rapid rise and fall (CK-MB) of biochemical markers of myocardial necrosis with at least one of the following: a) ischaemic symptoms; b) development of pathologic Q waves on the ECG; c) ECG changes indicative of ischaemia (ST segment elevation or depression); or d) coronary artery intervention (e.g., coronary angioplasty)
Eclampsia	Any episode of seizure antepartum, intrapartum or before postpartum discharge as follow-up beyond discharge is not possible
Require >50% oxygen for greater than one hour	Oxygen given at greater than 50% concentration based on local criteria for longer than 1 hour
Intubation other than for Caesarean section	Intubation by endotracheal tube
Severe breathing difficulty	Suspected pulmonary oedema where x-ray confirmation unavailable may be diagnosed by presence of chest pain or dyspnoea, crackles in the lungs and SaO_2 <90%
Pulmonary oedema	Clinical diagnosis with x-ray confirmation or requirement of diuretic treatment and SaO_2 <95%

Table S3 Development and Internal Validation Cohort Characteristics (n (%); median [interquartile range]

Variable	Development dataset (N=6633, 75%)	Internal Validation dataset (N=2210, 25%)	p-value*
Health system			
National *per capita* gross domestic product (USD)	43,586 [26,671-50,114]	43,586 [27,751-50,114]	0·798
National maternal mortality ratio (maternal deaths per 100,000 live births)	11 [11-15]	11 [11-15]	0·425
Demographics			
Race			<0·001
White	1935 (29·2%)	736 (33·3%)	
Asian	1989 (30·0%)	681 (30·8%)	
Black	1742 (26·3%)	589 (26·7%)	
Other	967 (14·6%)	204 (9·2%)	
Maternal age at expected date of delivery (years)	31 [26-36]	31 [27-36]	0·047
Nulliparous	3914 (59·0%)	1298 (58·7%)	0·822
Multiple pregnancy	494 (7·4%)	155 (7·0%)	0·510
Gestational age at eligibility (weeks)	35·8 [31·8-38·2]	35·6 [31·6-38·2]	0·769
Past and current medical and obstetrical history – no. (%)			
Cigarette smoking	861 (13·0%)	289 (13·1%)	0·913
Chronic hypertension	1126 (17·0%)	390 (17·6)	0·474
Pre-gestational renal disease	454 (6·8%)	161 (7·3%)	0·499
Pre-gestational diabetes	349 (5·3%)	122 (5·5%)	0·662
Gestational diabetes	773 (11·7%)	279(11·6%)	0·305
Symptoms on day of first assessment			
Nausea or vomiting	627 (9·5%)	368 (13·98%)	<0·001
Headache or visual disturbance	1977 (29·8%)	756 (34·2%)	<0·001
Right upper quadrant or epigastric pain	1218 (18·4%)	306 (13·8%)	<0·001
Chest pain or dyspnoea	679 (10·2%)	191 (8·6%)	0·029
Signs on day of first assessment			
Height(cm)	163 [157-168]	163 [157-168]	0·622
Weight (kg)	80·0 [69·9-93·0]	80·1 [69·4-93·8]	0·910
Systolic blood pressure (mm Hg)	150 [140-165]	150 [140-165]	0·963
Diastolic blood pressure (mm Hg)	96 [90-102]	97 [90-104]	0·133
Oxygen saturation less than 93%	49 (0·7%)	17 (0·8%)	0·887
Dipstick proteinuria (number of '+')	1 [1-2]	1 [1-2]	0·603
Laboratory tests – worst values on day of first assessment			
Haematocrit (%)	0·36 [0·34-0·38]	0·36 [0·34-0·39]	0·036
Total leucocyte count (x 10^9 per L)	10·5 [8·7-12·6]	10·4 [8·7-12·8]	0·437
Platelet count (x 10^9 per L)	206 [164-253]	210[167-254]	0·206

Variable	Development dataset (N=6633, 75%)	Internal Validation dataset (N=2210, 25%)	p-value*
Mean platelet volume (fL)	10·0 [8·9-11·2]	10·2 [9·4-11·2]	<0·001
Fibrinogen (g/L)	26·0 [24·5-28·2]	26·2 [24·3-29·0]	0·049
Activated partial thromboplastin time (sec)	26·4 [24·8-28·6]	26·6 [24·5-29·0]	0·085
Serum creatinine (µmol/L)	60 [51-71]	59 [49-70]	<0·001
Uric acid (mmol/)L	340 [286-402]	344 [284-402]	0·478
Aspartate transaminase (U/L	28 [21-39]	28 [21-39]	0·677
Alanine transaminase (U/L)	20 [13-32]	20 [13-31]	0·281
Albumin (g/L)	28 [23-32]	30 [25-33]	<0·001
Outcome			
In two days	450 (6·8%)	140 (6·3%)	0·491
In seven days	616 (9·3%)	197 (8·9%)	0·640
At any point	826 (12·5%)	257 (11·6%)	0·312

Table S4 Breakdown of Missing Data

Variable	Missing (%)									
	Vancouver N=1956	FINNPEC N=119	fullPIERS N=2011	miniPIERS N=2126	Oxford N=291	PETRA N=1244	PREP N=1096	Women with adverse outcome at any time after first assessment (N=1083)	Women without an adverse outcome (N=7760)	Total
Health system										
National per capita gross domestic product (USD)	0·00	0·00	0·40	0·47	1·03	0·00	0·00	1·94	0·00	0·24
National maternal mortality ratio (maternal deaths per 100,000 live births)	0·00	0·00	0·40	0·47	1·03	0·00	0·00	1·94	0·00	0·24
Demographics										
Race	26·12	0·84	19·69	1·69	30·58	0·64	0·55	10·53	12·02	11·84
Maternal age at expected date of delivery (years)	0·10	0·84	0·40	1·18	1·72	0·00	0·18	2·31	0·23	0·49
Nulliparous	0·15	0·84	0·40	0·52	1·03	0·00	0·00	2·12	0·04	0·29
Multiple pregnancy	0·00	0·84	0·40	0·52	1·03	0·24	0·00	2·03	0·05	0·29
Gestational age at eligibility (weeks)	0·10	0·00	0·00	0·61	0·69	0·00	0·00	0·18	0·19	0·19
Past and current medical and obstetrical history – no· (%)										
Cigarette smoking	1·48	2·52	5·47	2·45	2·75	0·40	0·00	4·06	2·10	2·34
Chronic hypertension	0·87	0·84	0·65	4·23	2·75	0·80	1·09	3·51	1·46	1·71
Pre-gestational renal disease	0·92	0·84	0·90	4·42	3·09	0·40	1·00	3·60	1·51	1·76
Pre-gestational diabetes	1·02	0·84	0·80	3·86	2·75	0·72	0·55	3·42	1·35	1·61
Gestational diabetes	1·64	0·84	0·99	2·54	2·06	0·24	0·00	3·14	1·06	1·31

Symptoms on day of first assessment										
Nausea or vomiting	3·68	22·69	0·90	2·96	13·06	97·27	77·28	24·93	25·84	25·73
Headache or visual disturbance	3·73	21·85	0·90	2·78	13·06	89·39	77·28	24·10	24·64	24·57
Right upper quadrant or epigastric pain	3·68	22·69	0·90	2·87	12·37	97·35	7·03	14·87	17·28	16·99
Chest pain or dyspnoea	3·73	22·69	61·16	2·78	13·06	91·96	77·28	35·27	39·12	38·65
Signs on day of first assessment										
Height(cm)	11·04	0·84	13·08	17·92	8·59	0·08	1·64	14·77	9·60	10·23
Weight (kg)	6·34	0·84	3·93	12·51	34·02	0·08	1·64	12·19	5·88	6·65
Systolic blood pressure (mm Hg)	8·08	21·85	2·83	3·25	14·43	13·67	77·37	20·87	14·74	15·49
Diastolic blood pressure (mm Hg)	8·08	21·01	2·83	3·34	14·43	13·67	77·37	20·78	14·77	15·50
Oxygen saturation (%)	0·00	0·00	0·00	0·00	0·00	0·00	90·15	12·93	10·93	11·17
Dipstick proteinuria (number of '+')	72·75	53·78	32·67	23·00	32·30	48·39	19·34	32·96	41·03	40·04
Laboratory tests – worst values on day of first assessment										
Haematocrit (%)	10·79	39·50	68·37	39·42	99·66	23·71	100·00	50·97	46·39	46·95
Total leucocyte count (x 10^9 per L)	9·92	39·50	10·84	58·00	13·40	100·00	23·27	37·21	36·43	36·53
Platelet count (x 10^9 per L)	10·33	39·50	10·99	37·82	13·40	24·04	23·18	24·01	20·70	21·10
Mean platelet volume (fL)	14·11	40·34	15·32	96·43	47·77	100·00	100·00	66·76	57·19	58·36
Fibrinogen (g/L)	22·44	92·44	29·04	75·16	89·69	86·01	61·68	56·23	53·21	53·58
Activated partial thromboplastin time (sec)	22·44	92·44	29·04	75·35	89·69	86·01	61·68	56·23	53·26	53·62
Serum creatinine (µmol/L)	18·35	77·31	13·72	52·54	12·71	31·19	22·90	30·29	28·25	28·50
Uric acid (mmol/L)	19·58	60·50	14·37	65·05	14·43	51·13	34·03	43·67	34·86	35·94
Aspartate transaminase (U/L)	18·66	99·16	16·41	66·60	72·85	31·03	76·19	51·62	39·99	41·41
Alanine transaminase (U/L)	18·40	41·18	12·78	54·09	35·74	31·35	25·73	36·84	28·26	29·31
Albumin (g/L)	26·12	79·83	32·02	95·91	15·81	100·00	25·09	57·99	54·46	54·89

Table S5 Sensitivity Analyses of Model Performance Excluding Organ Systems (N (%), likelihood ratio [95% confidence interval])

Risk Stratum*	PIERS-ML output (for outcomes within 2 d)	Hypertensive pregnant women in stratum	Hypertensive pregnant women with outcome within 2 d	Hypertensive pregnant women within risk stratum with outcome within 7 d	Hypertensive pregnant women within risk stratum with outcome at any time
	%	N (%)	N (%)	N (%)	N (%)
**Original PIERS-ML model (AUROC: 0·78 [0·73-0·82])**					
very low	0·0-0·5%	8 (0·7%)	0 (0·0%)	1 (12·5%)	1 (12·5%)
low	0·6-3·0%	321 (29·1%)	7 (2·2%)	13 (4·0%)	21 (6·5%)
moderate	3·1-18·6%	676 (61·3%)	34 (5·0%)	54 (8·0&)	76 (11·2%)
high	18·7-45·5%	87 (7·9%)	22 (25·3%)	24 (27·6%)	28 (32·2%)
very high	45·6-100%	11 (1·0%)	7 (63·6%)	7 (63·6%)	10 (90·9%)
**No platelet outcomes (AUROC: 0·76 [0·71-0·81])**					
very low	0·0-0·5%	8 (0·7%)	0 (0·0%)	1 (12·5%)	1 (12·5%)
low	0·6-3·0%	321 (29·1%)	7 (2·2%)	13 (4·0%)	21 (6·5%)
moderate	3·1-18·6%	676 (61·3%)	34 (5·0%)	54 (8·0%)	73 (10·8%)
high	18·7-45·5%	87 (7·9%)	22 (25·3%)	24 (27·6%)	27 (31·0%)
very high	45·6-100%	11 (1·0%)	7 (63·6%)	7 (63·6%)	7 (63·6%)
**No blood transfusion outcomes (AUROC: 0·78 [0·73-0·83])**					
very low	0·0-0·5%	8 (0·7%)	0 (0%)	1 (12·5%)	1 (12·5%)
low	0·6-3·0%	321 (29·1%)	5 (1·6%)	18 (2·5%)	21 (6·5%)
moderate	3·1-18·6%	676 (61·3%)	28 (4·1%)	46 (6·8%)	76 11·2%)
high	18·7-45·5%	87 (7·9%)	14 (16·1%)	14 (16·1%)	28 (32·2%)
very high	45·6-100%	11 (1·0%)	9 (81·8%)	9 (81·8%)	10 (90·9%)
**No renal outcomes (AUROC: 0·78 [0·73-0·82])**					
very low	0·0-0·5%	8 (0·7%)	0 (0·0%)	1 (12·5%)	1 (12·5%)
low	0·6-3·0%	321 (29·1%)	7 (2·2%)	13 (4·0%)	21 (6·5%)
moderate	3·1-18·6%	676 (61·3%)	34 (5·0%)	45 (8·3%)	76 (11·2%)
high	18·7-45·5%	87 (7·9%)	23 (26·4%)	25 (28·7%)	28 (32·2%)
very high	45·6-100%	11 (1·0%)	10 (90·9%)	10 (90·9%)	10 (90·9%)
**No platelet or blood transfusion outcomes (AUROC: 0·75 [0·69-0·80])**					
very low	0·0-0·5%	8 (0·7%)	0 (0·0%)	1 (12·5%)	1 (12·5%)
low	0·6-3·0%	321 (29·1%)	5 (1·6%)	8 (2·5%)	21 (6·5%)
moderate	3·1-18·6%	676 (61·3%)	26 (3·8%)	41 (6·1%)	76 (11·2%)
high	18·7-45·5%	87 (7·9%)	13 (14·9%)	13 (14·9%)	28 (32·2%)
very high	45·6-100%	11 (1·0%)	5 (45·5%)	5 (45·5%)	10 (90·9%)
**No platelet or renal outcomes (AUROC: 0·76 [0·71-0·81])**					
very low	0·0-0·5%	8 (0·7%)	0 (0·0%)	1 (12·5%)	1 (12·5%)
low	0·6-3·0%	321 (29·1%)	7 (2·2%)	13 (4·0%)	21 (6·5%)
moderate	3·1-18·6%	676 (61·3%)	32 (4·7%)	52 (7·7%)	76 (11·2%)
high	18·7-45·5%	87 (7·9%)	22 (25·3%)	24 (27·6%)	28 (32·2%)
very high	45·6-100%	11 (1·0%)	7 (63·6%)	7 (63·6%)	10 (90·9%)
**No blood transfusion or renal outcomes (AUROC: 0·77 [0·72-0·82])**					
very low	0·0-0·5%	8 (0·7%)	0 (0·0%)	1 (12·5%)	1 (12·5%)
low	0·6-3·0%	321 (29·1%)	6 (1·6%)	8 (2·5%)	21 (6·5%)
moderate	3·1-18·6%	676 (61·3%)	26 (3·8%)	44 (6·5%)	76 (11·2%)
high	18·7-45·5%	87 (7·9%)	14 (16·1%)	14 (16·1%)	28 (32·2%)
very high	45·6-100%	11 (1·0%)	9 (81·8%)	9 (81·1%)	10 (90·9%)
**No platelet, blood transfusion or renal outcomes (AUROC: 0·74 [0·68-0·80])**					
very low	0·0-0·5%	8 (0·7%)	0 (0·0%)	1 (12·5%)	1 (12·5%)
low	0·6-3·0%	321 (29·1%)	5 (11·6%)	8 (2·5%)	21 (6·5%)

moderate	3·1-18·6%	676 (61·3%)	24 (3·6%)	39 (5·8%)	76 (11·2%)
high	18·7-45·5%	87 (7·9%)	13 (14·9%)	13 (14·9%)	28 (32·2%)
very high	45·6-100%	11 (1·0%)	5 (45·5%)	5 (45·5%)	10 (90·9%)

AUROC, area under the receiver-operator characteristic; CI, confidence interval;-LR, negative likelihood ratio; +LR, positive likelihood ratio

* Risk strata determined by diagnostic test performances for first occurrence of any component of the primary combined adverse maternal outcome within two days: Very low risk: -LR <0·1; Low risk: -LR 0·1 - 0·2; Moderate risk: +LR <5·0 and -LR >0·2; High risk: +LR 5·0 – 10·0; Very high risk: +LR >10·0

Table S6 PIERS-ML Model Performance Using Alternative Machine Learning Strategies (N (%), likelihood ratio [95% confidence interval])

Risk Stratum*	PIERS-ML output (for outcomes within 2 d)	Hypertensive pregnant women in stratum	Hypertensive pregnant women with outcome within 2 d	Hypertensive pregnant women within risk stratum with outcome within 7 d	Hypertensive pregnant women within risk stratum with outcome at any time
	%	N (%)	N (%)	N (%)	N (%)
**Random forest with all variables (31 variables;** AUROC: 0·81 [0·76-0·85], AUPRC: 0·40)					
very-low	≤0·8%	21 (1·9%)	0 (0·0%)	1 (4·8%)	1 (4·8%)
low	0·9-4·6%	518 (46·9%)	12 (2·3%)	20 (3·9%)	33 (6·4%)
moderate	4·7-12·7%	419 (37·9%)	24 (5·7%)	40 (9·5%)	52 (12·4%)
high	12·8-21·3%	91 (8·2%)	16 (17·6%)	20 (22·0%)	21 (23·1%)
very-high	≥21·4%	56 (5·1%)	24 (42·9%)	26 (46·4%)	29 (51·8%)
**Random forest with recursive feature elimination (11 variables;** AUROC: 0·79 [0·75 - 0·83], AUPRC: 0·37)					
very-low	≤0·4%	11 (1·0%)	1 (9·1%)	1 (9·1%)	1 (9·1%)
low	0·5-3·1%	361 (32·7%)	5 (1·4%)	14 (3·9%)	22 (6·1%)
moderate	3·2-12·8%	605 (54·8%)	35 (5·8%)	53 (8·8%)	70 (11·6%)
high	12·9-29·0%	100 (9·0%)	19 (19·0%)	22 (22·0%)	25 (25·0%)
very-high	≥29·1%	28 (2·5%)	16 (57·1%)	17 (60·7%)	18 (64·3%)
**Random forest with Boruta feature selection (28 variables;** AUROC: 0·81 [0·76 - 0·85], AUPRC: 0·40)					
very-low	≤0·7%	18 (1·6%)	0 (0·0%)	2 (11·1%)	2 (11·1%)
low	0·8-4·7%	527 (47·7%)	12 (2·3%)	19 (3·6%)	32 (6·1%)
moderate	4·8-12·8%	406 (36·7%)	23 (5·7%)	39 (9·6%)	51 (12·6%)
high	12·9-22·8%	101 (9·1%)	17 (16·8%)	21 (20·8%)	22 (21·8%)
very-high	≥22·9%	53 (4·8%)	24 (45·3%)	26 (49·1%)	29 (54·7%)
**Random forest with Vita feature selection (31 variables;** AUROC: 0· 81 [0·77-0·85], AUPRC: 0·40)					
very-low	≤0·7%	14 (1·3%)	0 (0·0%)	1 (7·1%)	1 (7·1%)
low	0·8-4·7%	530 (48·0%)	12 (2·3%)	20 (3·8%)	33 (6·2%)
moderate	4·8-12·8%	409 (37·0%)	23 (5·6%)	40 (9·8%)	52 (12·7%)
high	12·9-21·5%	96 (8·7%)	16 (16·7%)	19 (19·8%)	20 (20·8%)
very-high	≥21·6%	56 (5·1%)	25 (44·6%)	27 (48·2%)	30 (53·6%)
**Gradient boosted tree with all variables** (AUROC: 0·82 [0·78-0·86], AUPRC: 0·40)					
very-low	≤ 1·3%	6 (0·5%)	0 (0·0%)	0 (0·0%)	0 (0·0%)
low	1·4-4·3%	575 (52·0%)	13 (2·3%)	22 (3·8%)	35 (6·1%)
moderate	4·4-10·8%	359 (32·5%)	20 (5·6%)	36 (10·0%)	49 (13·6%)
high	10·9-17·2%	85 (7·7%)	12 (14·1%)	15 (17·6%)	15 (17·6%)
very-high	≥ 17·3%	80 (7·2%)	31 (38·8%)	34 (42·5%)	37 (46·2%)
**Gradient boosted tree with variables of above average importance** (AUROC: 0·80 [0·76-0·85], AUPRC: 0·37)					
very-low	≤ 1·4%	7 (0·6%)	0 (0·0%)	0 (0·0%)	0 (0·0%)
low	1·5-2·9%	327 (19·6%)	6 (1·8%)	11 (3·4%)	18 (5·5%)
moderate	3·0-12·2%	631 (57·1%)	31 (4·9%)	51 (8·1%)	70 (11·1%)
high	12·3-23·8%	94 (8·5%)	19 (20·2%)	23 (24·5%)	24 (25·5%)
very-high	≥ 23·9%	46 (4·2%)	20 (43·5%)	22 (47·8%)	24 (52·2%)
**Artificial neural network with all variables** (AUROC: 0·78 [0·74-0·82], AUPRC: 0·26)					
very-low	NA	NA	NA	NA	NA
low	NA	NA	NA	NA	NA
moderate	≤ 15·0%	1027 (92·9%)	56 (5·5%)	85 (8·3%)	113 (11·0%)
high	15·1-47·1%	76 (6·9%)	19 (25·0%)	21 (27·6%)	22 (28·9%)
very-high	≥ 47·2%	2 (0·2%)	1 (50·0%)	1 (50·0%)	1 (50·0%)
**Artificial neural network with variables of above average importance** (AUROC: 0 78 [0·74-0·82], AUPRC: 0·28)					
very-low	NA	NA	NA	NA	NA

low	NA	NA	NA	NA	NA
moderate	≤ 15·8%	1034 (93·6%)	56 (5·4%)	85 (8·2%)	113 (10·9%)
high	15·7-45·1%	67 (6·1%)	18 (26·9%)	20 (29·9%)	21 (31·3%)
very-high	≥ 45·2%	4 (0·3%)	2 (50·0%)	2 (50·0%)	2 (50·0%)
**Least absolute shrinkage selection operator (LASSO)** (AUROC: 0·78 [0·74-0·83], AUPRC: 0·32)					
very-low	≤ 1·4%	20 (1·8%)	0 (0·0%)	0 (0·0%)	1 (5·0%)
low	1·5-2·0%	62 (5·6%)	0 (0·0%)	2 (3·2%)	3 (4·8%)
moderate	2·1-11·0%	877 (79·4%)	41 (4·7%)	65 (7·4%)	88 (10·0%)
high	11·1-48·7%	140 (12·7%)	31 (22·1%)	36 (25·7%)	40 (28·6%)
very-high	≥ 48·8%	6 (0·5%)	4 (66·7%)	4 (66·7%)	4 (66·7%)
**Bayesian model averaging** (AUROC: 0· 77 [0·73 - 0·82], AUPRC: 0·29)					
very-low	≤ 1·9%	33 (3·0%)	0 (0·0%)	0 (0·0%)	2 (6·1%)
low	2·0-2·3%	45 (4·1%)	1 (2·2%)	2 (4·4%)	3 (6·7%)
moderate	2·4-12·5%	924 (83·6%)	48 (5·2%)	76 (8·2%)	99 (10·7%)
high	12·6-22·8%	75 (6·8%)	13 (17·3%)	15 (20·0%)	18 (24·0%)
very-high	≥ 22·9%	28 (2·5%)	14 (50·0%)	14 (50·0%)	14 (50·0%)
**Ridge regression** (AUROC: 0· 78 [0·74 - 0·83], AUPRC: 0·31)					
very-low	≤ 2·3%	15 (1·4%)	0 (0·0%)	0 (0·0%)	1 (6·7%)
low	2·4-3·1%	95 (8·6%)	1 (1·1%)	2 (2·1%)	3 (6·7%)
moderate	3·2-10·8%	870 (78·7%)	41 (4·7%)	69 (7·9%)	93 (10·7%)
high	10·9-33·4%	118 (10·7%)	29 (24·6%)	31 (26·3%)	34 (28·8%)
very-high	≥ 33·5%	7 (0·6%)	5 (71·4%)	5 (71·6%)	5 (71·6%)

AUROC, area under the receiver-operator characteristic; CI, confidence interval;-LR, negative likelihood ratio; +LR, positive likelihood ratio

* Risk strata determined by diagnostic test performances for first occurrence of any component of the primary combined adverse maternal outcome within two days: Very low risk: -LR <0·1; Low risk: -LR 0·1 - 0·2; Moderate risk: +LR <5·0 and -LR >0·2; High risk: +LR 5·0 – 10·0; Very high risk: +LR >10·0

Table S7 Complete Case Analysis of the PIERS-ML and fullPIERS Models

Risk Stratum*	PIERS-ML outut (for outcomes within 2 d)	Hypertensive pregnant women in stratum	Hypertensive pregnant women with outcome within 2 d	Hypertensive pregnant women within risk stratum with outcome within 7 d	Hypertensive pregnant women within risk stratum with outcome at any time
	%	N (%)	N (%)	N (%)	N (%)
**Complete case analysis – PIERS-ML** (AUROC: 0·62 [0·43-0·81], AUPRC: 0·32)					
Very-low	≤ 0·5%	13 (3·7%)	0 (0%)	1 (7·7%)	1 (7·7%)
Low	0·6 - 3·0%	151 (43·0%)	5 (3·3%)	5 (3·3%)	6 (4·0%)
Moderate	3·1 - 18·6%	172 (49·0%)	6 (3·5%)	9 (5·2%)	12 (7·1%)
High	18·7 - 45·5%	12 (3·4%)	1 (8·3%)	1 (8·3%)	1 (8·3%)
Very-high	≥ 45·6%	3 (0·9%)	3 (100·0%)	3 (100·0%)	3 (100·0%)
**Complete case analysis - fullPIERS** (AUROC: 0·76 [0·64-0·88], AUPRC: 0·33)					
Very-low	≤ 0·5%	2 (0·6%)	0 (0%)	0 (0%)	0 (0%)
Low	0·6 - 3·0%	3 (0·9%)	0 (0%)	0 (0%)	0 (0%)
Moderate	3·1 - 18·6%	276 (78·6%)	8 (2·9%)	12 (4·3%)	15 (5·4%)
High	18·7 - 45·5%	56 (16·0%)	2 (3·6%)	2 (3·6%)	3 (5·4%)
Very-high	≥ 45·6%	14 (4·0%)	5 (35·7%)	5 (35·7%)	5 (35·7%)
**Complete case analysis – refitted fullPIERS** (AUROC: 0·64 [0·47-0·81], AUPRC: 0·26)					
Very-low	≤ 0·5%	0 (0·0%)	NA	NA	NA
Low	0·6 - 3·0%	17 (4·8%)	1 (5·9%)	1 (5·9%)	1 (5·9%)
Moderate	3·1 - 18·6%	318 (90·6%)	9 (2·8%)	13 (4·1%)	17 (4·8%)
High	18·7 - 45·5%	13 (3·7%)	3 (23·1%)	3 (23·1%)	3 (23·1%)
Very-high	≥ 45·6%	3 (0·9%)	2 (66·7%)	2 (66·7%)	2 (66·7%)
**Mean imputation – PIERS-ML** (AUROC: 0·79 [0·75-0·83], AUPRC: 0·38)					
Very-low	≤ 0·5%	9 (0·8%)	0 (0·0%)	1 (11·1%)	1 (11·1%)
Low	0·6- 3·0%	371 (33·6%)	10 (2·7%)	18 (4·9%)	28 (7·5%)
Moderate	3·1 - 18·6%	644 (58·3%)	34 (5·3%)	58 (8·4%)	71 (11·0%)
High	18·7 - 45·5%	70 (6·3%)	21 (30·0%)	23 (32·9%)	25 (35·7%)
Very-high	≥ 45·6%	11 (1·0%)	11 (100·0%)	11 (100·0%)	11 (100·0%)

AUROC, area under the receiver-operator characteristic; CI, confidence interval;-LR, negative likelihood ratio; +LR, positive likelihood ratio

* Risk strata determined by diagnostic test performances for first occurrence of any component of the primary combined adverse maternal outcome within two days: Very low risk: -LR <0·1; Low risk: -LR 0·1 - 0·2; Moderate risk: +LR <5·0 and -LR >0·2; High risk: +LR 5·0 – 10·0; Very high risk: +LR >10·0

PIERS-ML Data Sharing Statement

The PIERS data are de-identified participant-level data. As permitted by existing data sharing and collaboration agreements, the data will be available to academically-active entities (e.g., universities, NGOs, multilaterals), with the PIERS Principal Investigator (Peter von Dadelszen), or named delegate, as a named co-investigator, for the purposes of pregnancy hypertension-related research and within the limits of the informed consent obtained. Access will be through the Principal Investigator, or named delegate, contacted at 'pvd@kcl.ac.uk'. . A full data dictionary and all study documents will be available. Access will be through written application. When approved, a quote for the costs of preparing the data will be provided to the applicant.

By submitting an application form, the investigator agrees that s/he has read, understood and agrees to the terms and conditions below:

1. S/he is an academically-active researcher affiliated with an entity able to engage in a data

transfer agreement;

2. S/he warrants that the information entered is to the best of her/his knowledge full and correct;

3. S/he agrees that the Data Sharing Agreement will only be used for the specific project outlined in the application;

4. S/he represents that s/he has obtained the necessary approvals to transfer the data and/or receive the data under this Data Sharing Agreement;

5. S/he understands that the responses provided will form part of a legally-binding document;

6. S/he understands that the Agreement is not valid until a fully-executed copy, with signatures from all parties, is emailed to pvd@kcl.ac.uk; and

7. S/he understands that no modifications can be made to the Data Sharing Agreement and if modifications are made, the Data Sharing Agreement will be rendered invalid.

There is no pregnancy-specific repository for us to access.

Statistical supplement

Section S1: Methodology

Section S1·1: Machine learning

"Machine learning is an application of artificial intelligence (AI) that provides systems the ability to […] learn and improve from experience without being explicitly programmed." [1]

Unlike other mathematical modelling techniques, rather than explicitly programming a model which defines the form of the relationship between an outcome variable and potential predictors, machine learning methods are provided with many observations of the potential predictors, and in some cases, the outcome variable, referred to as the label. The machine learning method then learns the relationship from the data rather than from explicitly stated rules.

Machine learning can be supervised or unsupervised. In supervised learning the outcome data is labelled, and the aim of the model is to correctly identify the label of each observation in the data. The model is then able to compare the predicted label with the observed label to identify errors and make changes. After being trained on data with known labels, the machine learning algorithm can predict the label of new, unlabelled data; however, the accuracy of these predictions depends on many factors such as having suitable predictors, and the quality and quantity of the training data.

Section S1·2: Data Missingness

Table S4 shows the breakdown of missing data between women with and without adverse outcomes. Women who had an outcome had higher rates of missingness of GDP per capita, MMR, maternal age, parity, multiple pregnancy, all variables for past and current medical and obstetrical history, all variables for signs on day of admission bar dipstick proteinuria, haematocrit, platelet count, mean platelet volume, uric acid, AST, ALT, and albumin. They also had lower rates of missingness of right upper quadrant or epigastric pain, chest pain or dyspnoea, and dipstick proteinuria.

Data were not missing equally between studies (**Table S4**). Race was more likely to be missing in the Vancouver cohort, the fullPIERS cohort, and the Oxford cohort. Symptoms were rarely recorded within a day of first admission in the PETRA dataset and, except for right upper quadrant pain, in the PREP dataset. Blood pressure and oxygen saturation were missing for >70% on day of admission in the PREP data; however, blood pressure was often recorded after day of admission, with 90% of women in the PREP data having blood pressure recorded on or the day after of admission. The highest rate of missingness was reported for the laboratory tests, some tests missing entirely from some datasets. Fibrinogen, activated partial thromboplastin time, aspartate transaminase and albumin were rarely measured in the FINNPEC data. Mean platelet volume and albumin were rarely measured in the miniPIERS data. In the Oxford data, haematocrit was not measured; fibrinogen and activated partial thromboplastin time were rarely measured. Total leucocyte count, mean platelet volume, and albumin were not measured, and fibrinogen and activated partial thromboplastin time were rarely measured in the PETRA data. Haematocrit and mean platelet volume were not measured in the PREP data. The fullPIERS data had the least amount of missing data.

While rates of missingness were different between datasets and not all datasets recorded all variables, we assumed that patients within all cohorts were similar enough in their presentation that their observed and missing values for each variable would not be significantly different, or any potential difference can be accounted for by the other observed variables (such as gestational age). Datasets were combined on this assumption to create the data for model development and internal validation.

Section S1·3: Missing Data Imputation

While some machine learning methods have built in missing data imputation methods, many do not; hence it is important to handle missing data before applying machine learning methods which also ensures consistent data is used by each method. To do this, we first had to determine the mechanism of missingness for each variable with missing data. The mechanism of missingness can be missing completely at random (MCAR) when the missingness is completely by chance, missing at random (MAR) when missingness is related to some observed data but there is no relation between the observed and the unobserved data, or missing not at random (MNAR), where the unobserved data determines the missingness. [2]

Missing not at random was ruled out for all variables based on trial protocols and clinical input; however, it should be noted that this is an assumption only and not a test for mechanism of missingness. Total bilirubin, urinary protein to creatinine ratio, international normalised ratio, and lactate dehydrogenase had >60% missingness and were excluded from consideration in our model.

To test if data in any given variable were missing at random, we created a corresponding missingness variable coded 0 if there is a value observed in the given variable and 1 if missing. We then performed a t-test or a chi-square test (dependent on data type) on the missingness variable with all other predictor variables. If any of these tests is significant, the mechanism of missingness for the given variable would be missing at random. [3] All variables had at least one significant t- or chi-square test, meaning that all variables were missing at random.

Multiple imputation is generally suggested to be used for larger amounts of missing data; however, whether there is an amount of missingness that is too much for imputation in general is debatable. Previously it has been suggested that "too large" (such as 40% [4] or 60% [5]) amount of missing data should not be imputed; however, recent studies [6,7] suggest that variables with large amount of missingness can be imputed with minimal bias, given a large enough number of well defined imputations. We decided not to impute variables missing for at least 60% of patients within a day of first admission. As we had a mixture of numeric and categorical predictor variables, multiple chained random forests was selected as the method of imputation. We used the missRanger R package for imputation, imputing all predictor variables with missing data under 60%, using all available predictor variables as auxiliary variables.

Derived from Bodner's simulation study,[8] a rule of thumb for determining the number of imputations is to use at least the percentage of incomplete cases or more [9]. In our case, as ≈19% of all data were missing, development and validation datasets were imputed 20 times. To assess the impact of imputation on our model performance, we conducted a complete cases analysis the results of which are summarised in this document.

Section S1·4: Random forest

Random forest is a supervised machine learning ensemble method that grows several classification/regression trees in order to compensate for overfitting and bias and derive a more accurate prediction model. We used the caret R package with 10-fold cross-validation using the "rf" method to fit a random forest model on each imputed dataset. These random forest models were then combined into one ensemble model using the caretEnsemble R package.

On each of the imputed datasets, the caretList function was used to fit a single model using the "rf" random forest method of 500 trees, resulting in 20 caretList objects each with 1 element, which was a random forest model. The caretList objects were converted into simple lists and combined to create a list with 20 elements, each being a random forest model. This list was finally converted back into a caretList object, which then behaves as a single model, just as a caretList object with multiple models created on the same dataset using different modelling methods would.

While random forests, just like any other machine learning algorithm, can overfit, increasing the number of trees in a random forest does not increase the risk of overfitting. Additionally, due to the nature of the growing process of a random forest, while it uses all available variables, variables that do not significantly improve the predictive performance are given an importance of or close to zero. Variables are assigned an importance based on their mean decrease in the Gini index. Variables with a high mean decrease in Gini index have a large effect on the outcome while variables with a mean decrease in Gini index close to zero have little to no effect on the outcome.

This means that while all available variables are used to grow the random forest, some variables may be redundant and thus could be removed without significantly affecting model performance. As random forests provide us with a way to assess a variable's importance in a model, we can use variable selection methods and model performance assessment to remove predictor variables and identify the model with the lowest number of variables that still provides accurate predictions.

We selected the variables for our final model by first growing a random forest model using all variables, ranking them by importance and choosing only variables with above average importance. This is simply done by calculating the mean of the MeanDecreaseGini (the mean decrease in the Gini index) of all variables and selecting variables for the new forest only if their MeanDecreaseGini is greater than or equal to this mean.

In a random forest, variables are assigned an importance based on their mean decrease in the Gini index, a measure of node impurity. For each variable, the Gini index of node *k* for binary classification is calculated in each tree with Equation 1.

$$G(k) = 2 * p_0 * p_1 \qquad \textit{Equation 1}$$

where p_0 is the proportion of the data points in the node having no outcomes, and p_1 is the proportion of data points in the node with an outcome. [10] However, since in a random forest each tree is trained using a random subset of the data and the available variables, not the same proportion of observations will reach the node where the variable is used in the different trees. Hence, for random forests we measure the variable's total decrease in node impurity that occurs when a new split is created in a tree based on the values of a variable, and multiply it by the proportion of observations that reach the variable's node in the tree.

The mean of these weighted values is the Mean Decrease in Gini. The decrease in the Gini index or node impurity is calculated by Equation 2.

$$\Delta G = G(k) - \sum_{l=1}^{2} p_l G(k_l) \qquad \text{Equation 2}$$

Where $G(k)$ is the Gini index of the kth node, k_1 and k_2 are the nodes created by the split at the kth node, and $p_l = \frac{n_{k_l}}{n_k}$ for $l = \{1,2\}$ is the proportion of the population of the kth node split to the ${k_1}^{th}$ and ${k_2}^{th}$ nodes respectively. [11]

While we do not have access to beta coefficients in a random forest to understand the patterns of how each variable contributes to the outcome, we can calculate Shapley values for each individual prediction. Shapley values explain the contribution of each variable value to the difference of the predicted probability from the baseline for each prediction. The sum of Shapley values for a prediction is the difference between the average prediction and the current prediction. A positive Shapley value increases the predicted probability, a negative value decreases it and a value of zero does not change the predicted probability from the baseline.

Section S1·5: Risk groups

Using the PIERS-ML model, risk predictions were made on 12.5% of the data held out from model development. Following this, all possible predicted probabilities (to one decimal point) were tested

as a threshold by classing predictions greater than the threshold as predicted to have an outcome and predicted probabilities less than the threshold as predicted to not have an outcome. Positive and negative likelihood ratios were measured and stored for each threshold.

		Observed outcome	
		Yes	No
Predicted outcome	Yes	a	b
	No	c	d

Likelihood ratios are the probability of the predicted outcome to be observed in a patient with the same observed outcome, compared to a patient with the opposite observed outcome. More specifically, LR+ is the likelihood of a positive outcome predicted for a patient with a positive observed outcome, compared to a patient with a negative observed outcome, and LR- is the likelihood of a negative outcome predicted for a patient with a negative observed outcome, compared to a patient with a positive observed outcome. [12]

$$LR+= \frac{\text{sensitivity}}{1-\text{specificity}}$$

$$LR-= \frac{1-\text{sensitivity}}{\text{specificity}}$$

$$\text{sensitivity} = \frac{a}{a+c}$$

$$\text{specificity} = \frac{d}{d+b}$$

To select the lower probability threshold for the very high group, all possible predicted probabilities were with a positive likelihood ratio greater than or equal to 10 were selected. Any of these could arbitrarily be used as the threshold, we have selected to use the lowest value to maximize the number of patients classed into this risk group.

Next, possible probability thresholds greater than or equal to the very high group threshold were removed, and the selection process was repeated to find the threshold for the high risk group, this time looking for a positive likelihood ratio of 5 or greater. The lowest such value was selected.

For the very low risk group, probability thresholds less than the threshold for the high risk group, with negative likelihood ratio of 0.1 or less were selected, and the highest such value was used as the upper probability threshold for the very low risk group. Finally, for the low risk group, values greater than the very low risk group threshold, smaller than the high risk group threshold, with negative likelihood ratios of 0.2 or less were selected, and the highest value was used as the upper probability threshold for the low risk group.

Section S2: Results

*Section S2·1: Comparison of models (**Table S6**)*

Random forest was chosen as the best performing model. However, multiple modelling methods were tested, including gradient boosted trees, artificial neural networks, LASSO and ridge regression, and Bayesian model averaging.

LASSO (least absolute shrinkage and selection operator) and ridge regression are both penalised regression methods. Penalised regression works by adding a regularisation element controlled by a tuning parameter (λ) to the loss function when estimating the beta coefficients (β_j) of the predictor variables. There are two types of regularisation [13]:

- L1 regularisation, used by LASSO, where the regularisation element is $\lambda \sum_{j=1}^{p} |\beta_j|$, which penalises the regression model in a way that reduces the coefficients of variables that are not significant predictors of the outcome to 0; and
- L2 regularisation, used by ridge regression, where the regularisation element is $\lambda \sum_{j=1}^{p} \beta_j^2$, which penalises the regression model in a way that does not reduce the coefficients of any variables to 0.

LASSO and ridge regression were fitted using the caretEnsembe r package as the random forest models, using the “glmnet” method with alpha=1 and alpha=0, respectively

Gradient boosted trees, similarly to random forests, are also a method using a collection of classification trees. Unlike random forest, however, the trees used for gradient boosted tree models are grown with the intention to not be too complex, and generally smaller. These trees are also chained to make a prediction, each subsequent tree adding or subtracting from the predicted probability. Hence, while for a random forest, the output of the model is the mean of the prediction of each individual tree, in a gradient boosted tree model, the output is the sum of the predictions.

Gradient boosted tree models were fitted using the caretEnsembe r package as the random forest models, using the “xgbTree” method. This method uses the xgboost package. Detailed explanation of gradient boosted trees and the method can be found in the xgboost documentation page https://xgboost.readthedocs.io/en/stable/tutorials/model.html

Artificial neural networks (ANNs) are connected layers of nodes (neurons), replicating the neural networks of the brain. The models start with an input layer, where each node is a predictor variable, numbers are then fed forward to the subsequent layer by multiplying by a weight, adding a bias and applying some mathematical activation function, until reaching the output layer. The process is similar to a series of connected regression models [14] with the additional step of an activation function. The model fitting, or learning process of the algorithm, includes looking at each observation of the data, fitting adjusting the parameters of the model moving backwards from the output layer to minimise the error of the output, moving to the next observation and adjusting the parameters again, and so on [15].

ANN models were fitted using the caretEnsembe r package as the random forest models, using the “nnet” method.

Bayesian Model Averaging is a Bayesian method, unlike all other methods used, that works by estimating a posterior probability of each possible model given a prior probability, observed data and possible weights to be applied to the data. [16] for each model, a posterior probability is calculated [17], and each model is assessed and a decision on whether to include or exclude the model in the averaging process is made using Occam’s [18,19]. Using only the models that were not excluded in this step, the posterior distribution of coefficients is estimated [18]. Finally, models are ranked based on their posterior probabilities and the final model parameters are calculated by averaging over the top n models [20].

The bic.glm R function was used to create models using Bayesian model averaging.

The AUROC was very similar for all methods, since the outcome rate in the dataset is quite low; however, there was much bigger differences between the predicted risk groups. Random forest with variables of above average importance was selected to be the final model as we deemed it to have the best balance of the number of patients predicted in the very high and very low groups and the outcome rate in these groups.

*Section S2·2: Shapley values (**Figure 1A**)*

Shapley values come from cooperative game theory, created to calculate the contribution of each player in a cooperative game, by recording the pay-out of each different coalition of players[21]. In

machine learning, Shapley values similarly calculate the contribution of each feature to the prediction by calculating the average difference of the predicted probabilities for "coalitions" or value combinations of the features from the mean predicted probability[22]. Shapley values are calculated for each value of each feature. A Shapley value of 0 means that on average, the feature taking on the specified value does not change the predicted probability from the average, while a positive Shapley value indicates an increase in predicted probability, and a negative value indicates a decrease. **Figure 1A** shows the Shapley values for a single random forest on its corresponding validation dataset. The colour of each point represents the corresponding value of the feature, dark colour indicating a high value and light colour indicating a low value, while the height of the point clusters indicates the density of the points. The plot of these Shapley values shows any possible pattern between feature values and predicted probabilities. As the average predicted probability was low due to the low event rate, and most women had a low predicted probability, most values for each feature had a Shapley value close to zero. High values of serum creatinine, national MMR, aspartate transaminase and alanine transaminase, and low values of platelet count, oxygen saturation, national per capita GDP and haematocrit increased predicted probability. The biggest Shapley values were produced by a few observations of low oxygen saturation, while platelet count had the most non-zero Shapley values.

*Section S2·3: Model Calibration (**Figure 1C**)*

We assessed calibration using multiple methods. The first method used was calibration-in-the-large [23,24], comparing the mean predicted probability (0·074) to the prevalence of the outcome in the dataset (0·067). Perfect calibration-in-the-large is an absolute difference of 0, the larger the absolute difference, the worse the calibration is. The direction of difference is also informative - a mean predicted probability greater than the prevalence suggests that the model overestimates the risk, while a mean predicted probability smaller than the prevalence suggests that the model underestimates the risk [24]. As the absolute difference was 0·007 and mean predicted probability larger than the prevalence, we can see that the model slightly overestimates risk.

The second method used was the Cox calibration intercept and slope [23-26]. Perfect calibration is a Cox calibration intercept of 0 and slope of 1. As seen in plot B in Figure 1 of the main paper, the Cox Intercept for the model was 0·355, and the slope was 1·261. The Cox calibration intercept is also a measure of calibration-in-the-large, representing an overall bias in the predicted risk. The intercept of 0·355 shows an overestimation of risk, which is in line with the previous method of measuring calibration-in-the-large. The Cox calibration slope is a measure of spread, representing variability in the predicted probabilities. It should be noted that while the Cox calibration intercept can be interpreted on its own as a measure of calibration-in-the-large, the slope on its own does not measure calibration [26]. A slope of 1 can be observed along with both good and poor calibration-in-the-large. As the slope measures the spread of the predicted probabilities, a slope <1 can be interpreted as the predicted probabilities varying too much compared to the observed outcomes, and a slope >1 as the predicted probabilities not varying as much as the observed outcomes. The slope of 1·261 shows that the model predicts a smaller range of probabilities than expected. The highest predicted probability in the validation dataset was p=0·724 and the smallest p=0.002.

Cox calibration fits a straight line through the predicted probability and observed proportion of outcomes pairs, which is useful for overall calibration, but does not show us in what ranges of predicted probabilities our model performs worse. In addition, there is a chance of the model overestimating risk in one area and underestimating risk in another resulting in a good Cox calibration. We are able to assess if the model performs equally well or poorly across all predicted probabilities by obtaining a flexible calibration curve using loess (a nonparametric regression method) function [24], as seen in Figure 1B. The loess calibration curve shows that the model overestimates risk for the lower predicted probabilities, and underestimates risk for the higher predicted probabilities.

The final method used was the Spiegelhalter z test, derived from the Brier score (the mean squared difference between the predicted probability and the actual outcome). This method allows us to calculate a z statistic for calibration, which then can be tested to show if the model is statistically significantly improperly calibrated [25]. As the Spiegelhalter p-value was <0·05, the model was suboptimally calibrated; however, the LR-based risk strata placed women into appropriate risk groups.

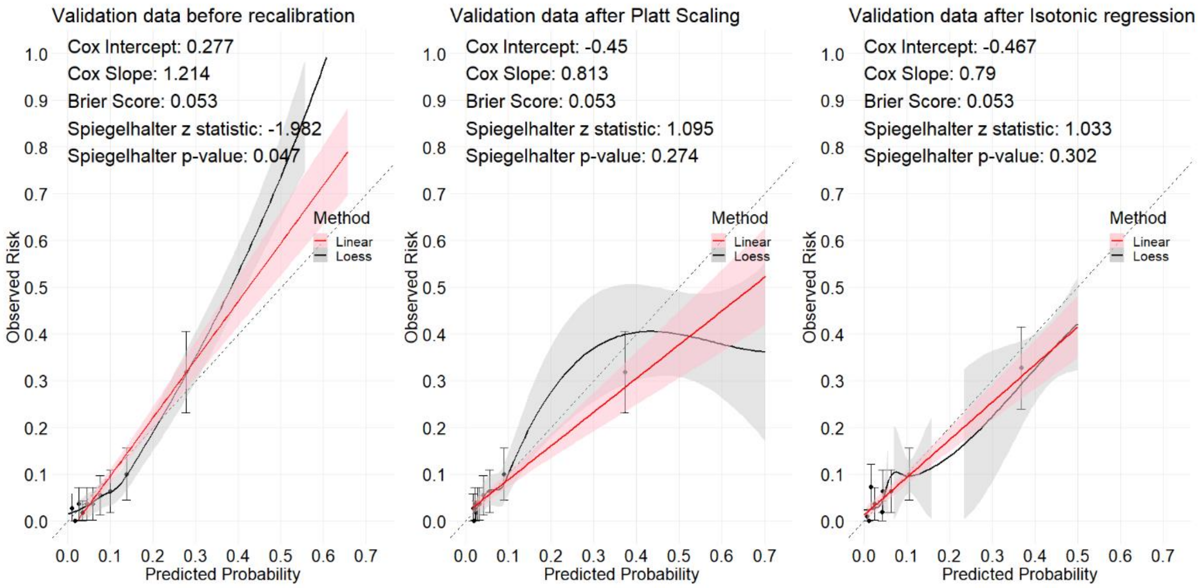

Figure S1 Calibration plots of original model (left), after Platt scaling (middle) and after Isotonic regression (right)

Despite calibration being sub-optimal, we had chosen to prioritise risk classification groups to give a more accurate prediction of risk. As the risk categories were determined based on likelihood ratios from the observed risk in the testing dataset, the selected threshold values account for the overestimation of small risk by moving the thresholds for very-low, low and moderate risk close together, and the underestimation of large risk by having wider ranges for the high and very-high risk groups.

To try to improve calibration, Platt scaling and isotonic regression was tested for re-calibration, but calibration did not improve (**Figure S1**). This could be due to not enough data.

*Section S2·4: Decision curve analysis (**Figure S2**)*

Decision curve analysis assesses clinical utility of a predictive model by calculating the Net Benefit of treating patients above each predicted probability threshold, compared to treating all patients or none. The net benefit is calculated for each probability threshold $p_{treshold}$ *by*

$$Net\ Benefit = \frac{TruePositiveCount}{n} - \frac{FlasePositiveCount}{n} * \frac{p_{treshold}}{1-p_{threshold}}$$

, where n is the total number of patients in the data, and the true and false positive counts for the model are determined by classing those with predicted probability greater than $p_{treshold}$ as predicted positive, and the rest as predicted negative [27]. To calculate the Net Benefit for treating all, all patients are classed as predicted positive for every threshold, while no patients are classed as predicted positive for the Net Benefit of treat none. Decision curve analysis assesses clinical utility by showing at which predicted probability thresholds would the model be more beneficial than treating all patients or none, if we were to treat only the patients with predicted probability above the specified threshold. While our PIERS-ML model was not intended to be used with a single threshold value to sort patients into a treatment or no treatment group, decision curve analysis was carried out to visualise the clinical utility of our method. The model had greater Net Benefit than treating all and treating none between the ~3% and 75% predicted probability thresholds. Risk groups are marked on the plot with dashed lines. Treating very high risk only, high and very high, and moderate, high and very high all have a greater net benefit than treating all or treating none, while treating all has a greater net benefit than including low, or low and very low groups.

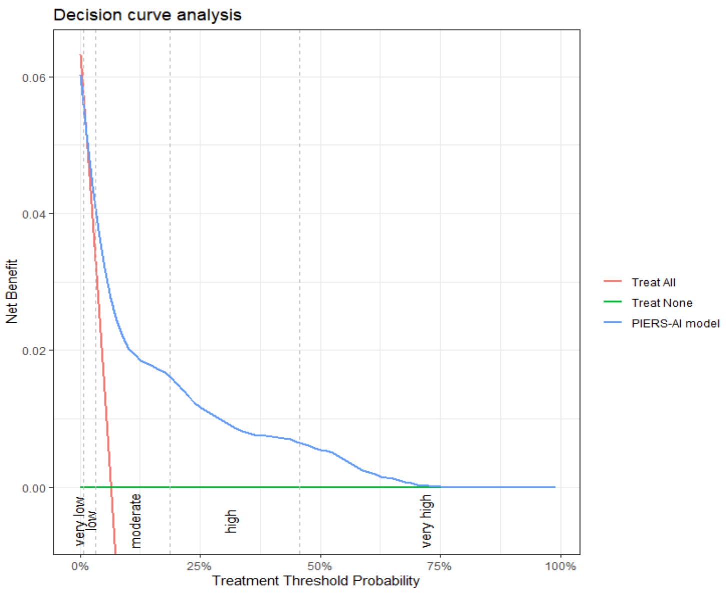

Figure S2 Decision curve analysis

*Section S2·5: Precision-recall curve (**Figure 1E**)*

While ROC curves plot sensitivity against 1-specificity for each possible predicted probability threshold, precision-recall curves plot precision (number of true positives/number of all positive predictions) against recall/sensitivity (number of true positives/number of all positive observations) for all thresholds. High precision means that the model makes few false positive predictions, while high recall means that the model can identify a large proportion of all positive instances. Similarly to ROC curves, precision-recall curves assess the performance of binary classification. However, unlike ROC curves, precision-recall curves are more accurate and more sensitive to change in performance when the outcome rate is low. This is because when the outcome rate is low, it is possible to get high AUROC values just by predicting no outcome for all patients due to the high number of true negatives and low number of false negatives. In this case, a precision-recall plot may be more informative than an ROC plot as neither precision nor recall depend on the true negative value. As our data had a low outcome rate, a precision-recall plot was included in Figure 1, with the minimum thresholds for each risk group indicated in the plot (**Figure 1E**). The precision-recall curve of the PIERS-ML model on the validation data is very close to a straight line, meaning that there is no one probability threshold with both good precision and good recall. However, as this was anticipated we were not looking for a single cut-off point to create a treatment and a no treatment group, rather we looked to create multiple risk groups, where the high and very high risk groups have high precision to rule in an outcome, and the low and very low risk groups have high recall to rule out. Recall did not need to be high in the high and very high risk groups as not being predicted in these

groups was not necessarily ruling out an outcome, and recall did not need to be low in the low and very low risk groups as not being predicted into these groups was not necessarily ruling in an outcome due to the inclusion of a non-informative moderate risk group. As expected, the low risk groups covered low precision and high recall thresholds, and the very high group had high precision and low recall; however, the high risk group covered moderate to high precision and moderate to low recall.

*Section S2·6: Complete case analysis (**Table S7**)*

Of the 2210 women in the internal validation dataset, 351 had no missing values for any of the PIERS-ML or fullPIERS variables (Table S6). To test model performance with no imputations, the PIERS-ML model, the original fullPIERS model and the refitted fullPIERS model were all tested on these complete cases. The complete cases had a lower outcome rate (4·3% in 2 days, 5·4% in 7 days, 6·6% at any point) than the whole of the dataset (6·65, 9·1% and 12·2% respectively). This is in line with the previous observation that patients with an outcome were more likely to have missing values. The PIERS-ML model performed better on the complete observations than the imputed validation datasets.

*Section S2·7: Mean imputation (**Table S7**)*

While PIERS-ML model is intended and expected to be used with all variables available for a patient, to simulate a practical example of the use of the model for a patient with missing data, we applied mean imputation to the validation dataset. The mean of each variable was calculated in the development dataset, and missing values in the validation data were replaced with the corresponding mean values. Both the number of patients predicted into each group and the number of outcomes per group were very similar between the original validation and the validation with mean imputation.

References

1. What is Machine Learning? A definition - Expert System. March 2020 (https://expertsystem.com/machine-learning-definition/).
2. Mack C, Su Z, Westreich D. AHRQ Methods for Effective Health Care. Managing Missing Data in Patient Registries: Addendum to Registries for Evaluating Patient Outcomes: A User’s Guide, Third Edition. Rockville (MD): Agency for Healthcare Research and Quality (US); 2018.
3. Grace-Martin K. How to Diagnose the Missing Data Mechanism - The Analysis Factor. 2013.
4. Jakobsen JC, Gluud C, Wetterslev J, Winkel P. When and how should multiple imputation be used for handling missing data in randomised clinical trials – a practical guide with flowcharts. BMC Medical Research Methodology 2017;17(1):162. DOI: 10.1186/s12874-017-0442-1.
5. Swalin A. How to Handle Missing Data - Towards Data Science. Medium 2018 (In English).

6. Lee JH, Huber JC, Jr. Evaluation of Multiple Imputation with Large Proportions of Missing Data: How Much Is Too Much? Iranian journal of public health 2021;50(7):1372-1380. (In eng). DOI: 10.18502/ijph.v50i7.6626.

7. Madley-Dowd P, Hughes R, Tilling K, Heron J. The proportion of missing data should not be used to guide decisions on multiple imputation. Journal of Clinical Epidemiology 2019;110:63-73. DOI: https://doi.org/10.1016/j.jclinepi.2019.02.016.

8. Bodner TE. What improves with increased missing data imputations? Structural equation modeling: a multidisciplinary journal 2008;15(4):651-675.

9. White IR, Royston P, Wood AM. Multiple imputation using chained equations: Issues and guidance for practice. Statistics in Medicine 2011;30(4):377-399. (https://doi.org/10.1002/sim.4067). DOI: https://doi.org/10.1002/sim.4067.

10. Brownlee J. Classification And Regression Trees for Machine Learning. August 15, 2020 (https://machinelearningmastery.com/classification-and-regression-trees-for-machine-learning/).

11. Menze BH, Kelm BM, Masuch R, et al. A comparison of random forest and its Gini importance with standard chemometric methods for the feature selection and classification of spectral data. BMC Bioinformatics 2009;10(213) (In eng).

12. Likelihood Ratios — Centre for Evidence-Based Medicine (CEBM), University of Oxford. 2020 (Web Page).

13. Nagpal A. L1 and L2 Regularization Methods. 2017.

14. Education IC. What are Neural Networks? 2021-08-03 (https://www.ibm.com/cloud/learn/neural-networks).

15. Naviani A. ANN (Artificial Neural Network) Models in R: Code & Examples on How to Build Your NN. 2021.

16. Raftery A, Hoeting J, Volinsky C, Painter I, Yeung KY. BMA: Bayesian Model Averaging. 2020.

17. Madigan D, Raftery AE. Model Selection and Accounting for Model Uncertainty in Graphical Models Using Occam's Window. Journal of the American Statistical Association 1994;89(428):1535-1546. DOI: 10.2307/2291017.

18. Raftery AE, Madigan D, Hoeting JA. Bayesian Model Averaging for Linear Regression Models. https://doiorg/101080/01621459199710473615 2012 (research-article) (In en). DOI: Journal of the American Statistical Association, Vol. 92, No. 437, March 1997: pp. 179–191.

19. Raftery AE. Bayesian Model Selection in Social Research. Sociological Methodology 1995;25:111-163. DOI: 10.2307/271063.

20. Hinne M, Gronau QF, Bergh Dvd, Wagenmakers E-J. A Conceptual Introduction to Bayesian Model Averaging:. https://doiorg/101177/2515245919898657 2020 (research-article) (In en). DOI: 10.1177_2515245919898657.

21. Shapley LS. A Value for N-Person Games. Santa Monica, CA: RAND Corporation, 1952.

22. Molnar C. Interpretable Machine Learning: A Guide for Making Black Box Models Explainable. Interpretable Machine Learning: A Guide for Making Black Box Models Explainable. 2 ed2022.

23. Steyerberg EW, Vickers AJ, Cook NR, et al. Assessing the performance of prediction models: a framework for traditional and novel measures. Epidemiology (Cambridge, Mass) 2010;21(1):128-138. (In eng). DOI: 10.1097/EDE.0b013e3181c30fb2.

24. Van Calster B, McLernon DJ, van Smeden M, et al. Calibration: the Achilles heel of predictive analytics. BMC Medicine 2019;17(1):230. DOI: 10.1186/s12916-019-1466-7.

25. Huang Y, Li W, Macheret F, Gabriel RA, Ohno-Machado L. A tutorial on calibration measurements and calibration models for clinical prediction models. Journal of the American Medical Informatics Association 2020;27(4):621-633. DOI: 10.1093/jamia/ocz228.

26. Stevens RJ, Poppe KK. Validation of clinical prediction models: what does the “calibration slope” really measure? Journal of Clinical Epidemiology 2020;118:93-99. DOI: https://doi.org/10.1016/j.jclinepi.2019.09.016.

27. Vickers AJ, Elkin EB. Decision Curve Analysis: A Novel Method for Evaluating Prediction Models. Medical Decision Making 2006;26(6):565-574. DOI: 10.1177/0272989X06295361.
